# Indolocarbazole-Based Photo-Crosslinkable Hole-Transporting Layer for Efficient Solution-Processed Organic Light-Emitting Diodes

**DOI:** 10.3390/nano13131934

**Published:** 2023-06-25

**Authors:** Jeong Yong Park, Seon Lee Kwak, Hea Jung Park, Do-Hoon Hwang

**Affiliations:** 1 Department of Chemistry and Chemistry Institute for Functional Materials, Pusan National University, Busan 46241, Republic of Korea; pjy9189@naver.com (J.Y.P.); gsun0331@naver.com (S.L.K.); 2 Department of Biology and Chemistry, Changwon National University, Changwon 51140, Republic of Korea

**Keywords:** indolocarbazole, photo-crosslinking, hole-transporting material, organic light-emitting diodes

## Abstract

We designed and synthesized a new indolocarbazole-based polymer, poly(N,N-diphenyl(5,11-dihexylindolo[3,2,1-jk]carbazol-2-yl)amine) (PICA), for solution-processed organic light-emitting diodes (OLEDs). The highest occupied and lowest unoccupied molecular orbital energy levels of this polymer, −5.25 and −2.46 eV, respectively, are suitable for hole transport from the anode to the emissive layer. PICA was photo-crosslinked by UV irradiation with ethane-1,2-diyl bis(4-azido-2,3,5,6-tetrafluorobenzoate) (FPA) as the photoinitiator. Successful crosslinking was confirmed by a decreased intensity in the azide-stretching FT-IR peak and solvent test with toluene (a suitable solvent for PICA). The PICA film photo-crosslinked with 3 wt% FPA showed enhanced solvent resistance (90%) compared to the non-crosslinked neat PICA film (<20%). Moreover, OLED devices using PICA-based hole-transporting layers exhibited better device performance (EQE/LE/PE: 8.88%/12.97/8.12 in red devices, 10.84%/38.47 cd/A/25.06 lm/W in green devices) than those using poly-TPD:FPA. We demonstrated that the photo-crosslinked PICA can be applied as a hole-transporting layer in solution-processed OLEDs.

## 1. Introduction

Recently, organic light-emitting diodes (OLEDs) have become representative displays because of their high brightness and contrast, fast response, flexibility, high color purity, and low power consumption [1,2,3]. OLEDs show high device efficiency by adopting multiple organic thin layers consisting of hole and electron injection layers, hole and electron transport layers, and emissive layers [4,5,6,7]. In the OLED, holes and electrons generated at the anode and cathode, respectively, move to the emissive layer through the injection and the transport layers. Subsequently, light is generated through the recombination of the excitons. Vacuum sublimation is one of the major OLED device fabrication methods that has high device efficiency and stable device characteristics [6]. In contrast, the solution-processed OLED manufacturing strategy is attracting attention because it is favorable to large-area technologies such as inkjet, roll-to-roll printing, or spin casting [8,9,10,11]. A major problem in device fabrication through solution processing is that the lower organic layer can be damaged by solvent used in forming the upper layer. The dissolution or interfacial mixing makes it difficult to manufacture a multilayered thin film by adopting solution processing. This is one of the reasons that the solution-processed OLED device shows relatively lower device performances such as efficiency and lifetime than those of the vacuum-deposited OLED device. Many efforts are being made to create a thin film with high solvent resistance in order to prevent thin films from being damaged during the solution process [12,13,14,15,16,17,18,19,20,21,22].

An approach to create an insoluble network film through chemical crosslinking has been extensively studied as a method of increasing solvent resistance. Additionally, using an azide-based photo-crosslinking group in the side chain of a polymer is known as a good crosslinking method because of its excellent chemical stabilities and it does not significantly degrade the performance of the devices [23]. In particular, ethane-1,2-diylbis(4-azido-2,3,5,6-tetrafluorobenzoate) (FPA) has been used as a photo initiator and cross-linker, which has high efficiency of insertion to the alkyl position [24,25].

The terminal azide group is transformed into the active species, nitrene, by the loss of N_2_ upon relatively low-dose UV irradiation. The nitrene can be inserted into the alkyl position (C–H) to form a new covalent bond creating an insoluble network film. Our group also reported a blend of poly(bis-4-butylphenyl-N,N-bisphenyl)benzidine (poly-TPD) and FPA as a photo-crosslinkable hole-transporting layer (HTL) [26].

We have studied a new indolocarbazole-based photo-crosslinkable hole-transporting polymer with the proper HOMO level and alkyl side chains. Indolocarbazole derivatives have been studied as efficient and stable hole transport materials. The planar fused ring system with less ring strain and the strong electron donating ability of indolo[3,2,1-jk] carbazole is advantageous for hole transporting in organic thin-film transistors, perovskite solar cells, or dye-sensitized solar cells [27,28,29,30,31,32,33]. Taking the above into account, we develop an indolo[3,2,1-jk]carbazole-based novel polymer as a photo-crosslinkable HTL material with FPA photo initiator to achieve efficient solution-processed OLEDs.

**Scheme 1:**
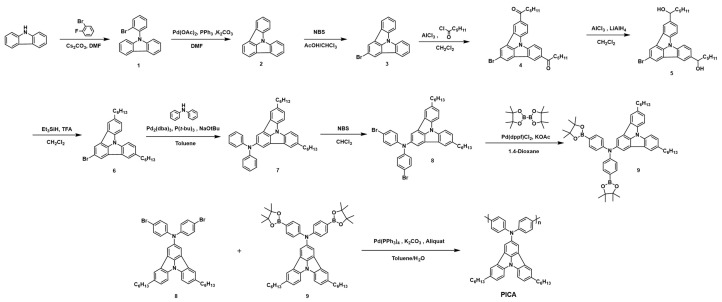
Synthesis of PICA, the new polymer for HTL.

In this work, we successfully synthesized an indolo[3,2,1-jk] carbazole-based new polymer, poly(N,N-diphenyl(5,11-dihexylindolo[3,2,1-jk]carbazol-2-yl)amine) (PICA), as a hole-transporting material for solution-processed OLEDs. In order to raise the deep highest occupied molecular orbital (HOMO) energy level of indolo[3,2,1-jk]carbazole (−5.81 eV) [34] to an energy level between PEDOT:PSS (−5.0 eV) and light-emitting layers (above −5.6 eV), an electron-rich diphenylamine group was introduced on the 2-position of indolo[3,2,1-jk]carbazole, which is suitable for use as an HTL material with hole injection. Two hexyl groups were substituted on 5- and 11-positions of indolo[3,2,1-jk]carbazole unit not only to improve the molecular solubility required during the synthetic process, but also to increase the chemical crosslinking sites. The synthetic route and chemical structure of the PICA are shown in Scheme 1. The detailed synthetic procedures are described in Section 3. To the best of our knowledge, this study is the first to use an indolo[3,2,1-jk]carbazole-containing polymer as a crosslinkable HTL material for solution-processed OLEDs. As a result, the photo-crosslinked PICA:FPA (3 wt%) exhibited superior device performances in the red and green OLED devices (EQE/LE/PE: 8.88%/12.97 cd/A/8.12 lm/W in the red devices, 10.84%/38.47 cd/A, 25.06 lm/W in the green devices) compared to those of the reference material, photo-crosslinked poly-TPD: FPA (3 wt%).

## 2. Materials and Methods

### 2.1. Materials

Carbazole, 1-bromo-2-fluorobenzene, cesium carbonate, aluminum chloride, potassium carbonate, potassium acetate triphenylphosphine, tetrabutylammonium bromide, N-bromosuccinimide, hexanoyl chloride, triethylsilane, trifluoroacetic acid, tris(dibenzylideneacetone)dipalladium, lithium aluminum hydride solution, tetrakis(triphenylphosphine)palladium, and bis(pinacolato)diboron, 4,4′,4-tris(carbazol-9-yl)triphenylamine (TCTA), 2,2′,2”-(1,3,5-Benzinetriyl)-tris(1-phenyl-1-H-benzimidazole (TPBi), 4,4′-bis(N-carbazolyl)-1,1′-biphenyl (CBP), poly(9-vinylcarbazole) (PVK) were purchased from Sigma-Aldrich, Alfa Aesar, and Tokyo Chemical Industry. They were used without further purification. Tris[2-(p-tolyl)pyridine]iridium(III) (Ir(mppy)_3_), iridium(III) bis[4-methyl-2-(3,5-dimethylphenyl)quinolinato-N,C2′]tetramethylheptadionate (Ir(mphmq)_2_(tmd)), poly-TPD, and FPA were synthesized according to reported methods [35,36,37,38,39,40].

### 2.2. Synthesis

Synthesis of 9-(2-bromophenyl)-9H-carbazole (1)

Carbazole (20.0 g, 119.6 mmol), cesium carbonate (97.4 g, 299.0 mmol), and 1-bromo-2-fluorobenzene (27.2 g, 155.5 mmol) were dissolved in dimethyl sulfoxide (400 mL). The mixture was refluxed for 12 h. The reaction mixture was then cooled to room temperature, extracted with dichloromethane, and the organic layer was dried over magnesium sulfate. After removing the solvent under reduced pressure, the crude product was purified using silica gel column chromatography (dichloromethane/hexane, 1:5 *v*/*v*) to afford compound 1 (30.2 g, 79%). ^1^H-NMR (CDCl_3_, 400 MHz, ppm): δ 8.18 (d, J = 7.6 Hz, 2H), 7.89 (dd, J = 8 Hz, 1.2 Hz, 1H), 7.57–7.49 (m, 2H), 7.45–7.40 (m, 3H), 7.32(td, J = 6.8 Hz, 0.8 Hz, 2H), 7.09 (d, J = 8.4 Hz, 2H). ^13^C-NMR (CDCl_3_, 100 MHz, ppm): δ 141.0, 134.3, 131.3, 130.3, 129.0, 126.2, 124.0, 123.4, 120.5, 120.2, 110.2. HRMS (ESI): *m*/*z* calcd. for C_18_H_12_BrN [M + H]^+^ 322.0226, found 322.0207.

Synthesis of indolo[3,2,1-jk]carbazole (2)

Compound 1 (25.0 g, 53.9 mmol), palladium acetate (1.7 g, 7.7 mmol), potassium carbonate (26.8 g, 193.8 mmol), triphenylphosphine (8.1 g, 30.8 mmol), and tetrabutylammonium bromide (0.7 g, 2.3 mmol) were dissolved in dimethylformamide (300 mL). The mixture was refluxed for 6 h. After the reaction mixture cooled to room temperature, it was extracted with chloroform and dried over magnesium sulfate. After removing the solvent under reduced pressure, the crude product was purified using silica gel column chromatography (dichloromethane/hexane, 1:7 *v*/*v*) to afford compound 2 (14.3 g, 77%). ^1^H-NMR (CDCl_3_, 400 MHz, ppm): δ 8.17 (dt, J = 8.0 Hz, 0.8 Hz, 2H), 8.07 (d, J = 7.6 Hz, 2H), 7.94 (dt, J = 8.0 Hz, 0.8 Hz, 2H), 7.62–7.55 (m, 3H), 7.39 (td, J = 7.6Hz, 0.8 Hz, 2H). ^13^C-NMR (CDCl_3_, 100 MHz, ppm): δ 143.8, 138.7, 130.1, 126.8, 123.1, 122.8, 121.7, 119.4, 118.5, 112.2. HRMS (ESI): *m*/*z* calcd. for C_18_H_11_N [M + H]^+^ 242.0964, found 242.0965.

Synthesis of 2-bromoindolo[3,2,1-jk]carbazole (3)

Compound 2 (13.0 g, 53.9 mmol) was dissolved in a mixture of chloroform (150 mL) and acetic acid (150 mL). N-bromosuccinimide was added to the mixture at 0 °C. After the mixture was heated at 50 °C for 6 h, it was extracted with chloroform and dried over magnesium sulfate. After removing the solvent under reduced pressure, the crude product was washed with glacial acetic acid to obtain compound 3 as a pure product (6.2 g, 36%). ^1^H-NMR (CDCl_3_, 400 MHz, ppm): δ 8.17 (s, 2H), 8.10 (dd, J = 7.6 Hz, 0.8 Hz, 2H), 7.91 (dd, J = 7.6 Hz, 0.8 Hz, 2H), 7.62 (td, J = 7.6 Hz, 0.8 Hz, 2H), 7.39 (td, J = 7.6 Hz, 0.8 Hz, 2H). ^13^C-NMR (CDCl_3_, 100 MHz, ppm): δ 142.0 139.1, 129.3, 127.6, 123.5, 122.5, 122.2, 119.8, 115.9, 112.5. HRMS (ESI): *m*/*z* calcd. for C_18_H_11_BrN [M + H]^+^ 320.0069, found 320.0040.

Synthesis of 1,1′-(2-bromoindolo[3,2,1-jk]carbazole-5,11-diyl) bis(hexan-1-one) (4)

AlCl_3_ (6.5 g, 48.2 mmol) was added to a dry two-neck flask, followed by anhydrous CH_2_Cl_2_ (100 mL). Then, hexanoyl chloride (48.2 mmol, 7.3 g) was added slowly, and the mixture was stirred for 20 min at 0 °C. After adding compound 3 (6.2 g, 19.3 mmol), the mixture was slowly warmed up to room temperature and stirred for 12 h. The mixture was then cooled to 0 °C and quenched on ice. The mixture was extracted with chloroform and dried over magnesium sulfate. After removing the solvent under reduced pressure, the crude product was recrystallized in dichloromethane/MeOH to give compound 4 as a pure product (8.1 g, 82%). ^1^H-NMR (CDCl_3_, 400 MHz, ppm): δ 8.60 (d, J = 1.6 Hz, 2H), 8.22 (dd, J = 8.4 Hz, 1.6 Hz, 2H), 8.10 (s, 2H), 7.80 (d, J = 8.4 Hz, 2H), 3.10 (t, J = 7.6 Hz, 4H), 1.89–1.82 (m, 4H), 1.47–1.43 (m, 8H), 0.98–0.95 (m, 6H). ^13^C-NMR (CDCl3, 400 MHz, ppm): δ 199.2, 142.6, 140.1, 131.8, 129.0, 127.9, 123.4, 122.9, 119.1, 117.0, 111.8, 38.8, 31.8, 24.2, 22.8, 14.2. HRMS (ESI): *m*/*z* calcd. for C_30_H_30_BrNO_2_ [M + H]^+^ 516.1543, found 516.1534.

Synthesis of 1,1′-(2-bromoindolo[3,2,1-jk]carbazole-5,11-diyl) bis(hexan-1-ol) (5)

AlCl_3_ (37.1 mmol, 4.9 g) was added to a dry one-necked flask, followed by anhydrous tetrahydrofuran (150 mL). LiAlH_4_ solution (47 mL, 2.0 M in THF) was added slowly and stirred for 20 min at 0 °C. Compound 4 (8.1 g, 15.7 mmol) was added, and the mixture was slowly warmed up to room temperature and stirred for 3 h. The mixture was then cooled to 0 °C and quenched on ice. The mixture was extracted with chloroform and dried over magnesium sulfate. After removing the solvent under reduced pressure, the crude product was washed with MeOH to obtain compound 5 as a pure product (7.8 g, 96%). ^1^H-NMR (CDCl_3_, 400 MHz, ppm): δ 8.10 (s, 2H), 8.01 (d, J = 1.6 Hz, 2H) 7.77 (d, J = 8.4 Hz, 2H) 7.53 (dd, J = 8.4 Hz, 1.6 Hz, 2H), 4.87 (t, J = 6.8 Hz, 4H), 1.93–1.81 (m, 4H), 1.57–1.43 (m, 8H), 0.89–0.86 (m, 6H). ^13^C-NMR (CDCl_3_, 100 MHz, ppm): δ 142.8, 139.1, 138.4, 129.4, 125.7, 122.6, 121.0, 119.8, 115.8, 112.1, 75.0, 39.7, 31.9, 25.8, 22.8, 14.2. HRMS (ESI): *m*/*z* calcd. for C_30_H_34_BrNO_2_ [M + H]^+^ 520.1846, found 520.1686.

Synthesis of 2-bromo-5,11-dihexylindolo[3,2,1-jk]carbazole (6)

Compound 5 (7.0 g, 13.4 mmol) was dissolved in dichloromethane (250 mL), and then trifluoroacetic acid (6.1 g, 53.2 mmol) was added dropwise at 0 °C. Subsequently, triethylsilane (6.1 g, 53.2 mmol) was added dropwise at 0 °C, and the mixture was slowly warmed up to room temperature and stirred for 6 h. Then, the mixture was extracted with dichloromethane and dried over magnesium sulfate. The crude product was purified by silica gel column chromatography (dichloromethane/hexane, 1:8 *v*/*v*) and reprecipitated using hexane to afford compound 6 as a pure product (5.1 g, 78%). ^1^H-NMR (CDCl_3_, 400 MHz, ppm): δ 8.12 (s, 2H), 7.87 (d, J = 1.6 Hz, 2H) 7.76 (d, J = 8.4 Hz, 2H), 7.39 (dd, J = 8.0 Hz, 1.6 Hz, 2H), 2.81 (t, J = 7.6 Hz, 4H), 1.74–1.70 (m, 4H), 1.42–1.31 (m, 8H), 0.93–0.88 (m, 6H). ^13^C-NMR (CDCl_3_, 100 MHz, ppm): δ 142.6, 137.5, 136.8, 129.3, 128.0, 123.1, 122.2, 119.8, 115.5, 111.9, 36.2, 32.2, 32.0, 29.2, 22.8, 14.3. HRMS (ESI): *m*/*z* calcd. for C_30_H_34_BrN [M + H]^+^ 488.1947, found 488.1885.

Synthesis of 5,11-dihexyl-N,N-diphenylindolo[3,2,1-jk]carbazol-2-amine (7).

Compound 6 (4.0 g, 8.2 mmol), diphenyl amine (1.7 g, 9.8 mmol), tris(dibenzylideneacetone)dipalladium (0.3 g, 0.3 mmol), sodium tert-butoxide (2.4 g, 24.6 mmol), and tri-tert-butylphosphine solution (0.1 g, 0.6 mmol) were dissolved together in toluene under N_2_ atmosphere and refluxed overnight. After cooling to room temperature, the reaction mixture was extracted with ethyl acetate and dried over magnesium sulfate. The crude product was purified using silica gel column chromatography (dichloromethane/hexane, 1:8 *v*/*v*) and reprecipitated using diethyl ether to afford compound 7 as a yellow powder (3.7 g, 78%). ^1^H-NMR (CDCl_3_, 400 MHz, ppm): δ 7.88 (s, 2H), 7.82 (d, J = 1.6 Hz, 2H), 7.36 (dd, J = 8.4 Hz, 1.6 Hz, 2H), 7.25–7.22 (m, 4H), 7.14–7.12 (m, 4H), 7.00–6.95 (m, 2H), 2.78 (t, J = 7.6 Hz, 4H), 1.70–1.65 (m, 4H), 1.36–1.30 (m, 8H), 0.90–0.87 (m, 6H). ^13^C-NMR (CDCl_3_, 100 MHz, ppm): δ 149.4, 143.6, 142.2, 137.7, 136.5, 130.0, 129.2, 127.5, 123.2, 122.7, 121.6, 120.3, 119.3, 111.9, 36.2, 32.2, 32.0, 29.1, 22.8, 14.3. HRMS (ESI): *m*/*z* calcd. for C_42_H_44_N_2_ [M + H]^+^ 577.3577, found 577.3576.

Synthesis of N,N-bis(4-bromophenyl)-5,11-dihexylindolo[3,2,1-jk]carbazol-2-amine (8).

Compound 7 (3.5 g, 6.1 mmol) was dissolved in chloroform, and then N-bromosuccinimide (2.5 g, 13.9 mmol) was added dropwise at 0 °C. The mixture was stirred at 0 °C for 3 h and then extracted with dichloromethane and dried over magnesium sulfate. The crude product was purified using silica gel column chromatography (dichloromethane/hexane, 1:6 *v*/*v*) and reprecipitated using diethyl ether and dichloromethane to obtain compound 8 as a yellow powder (3.06 g, 69%). ^1^H-NMR (CDCl_3_, 400 MHz, ppm): δ 7.83 (d, J = 1.6 Hz, 2H), 7.80 (s, 2H), 7.78 (d, J = 8.4 Hz, 2H), 7.38 (dd, J = 8.0 Hz, 1.6 Hz, 2H), 7.33–7.31 (m, 4H), 7.00–6.97 (m, 4H), 2.79 (t, J = 7.6 Hz, 4H), 1.70–1.68 (m, 4H), 1.38–1.30 (m, 8H), 0.90–0.87 (m, 6H). ^13^C-NMR (CDCl_3_, 100 MHz, ppm): δ 148.0, 142.6, 142.3, 137.8, 136.7, 132.3, 129.8, 127.8, 124.2, 123.2, 119.9, 119.5, 114.3, 111.9, 36.2, 32.2, 32.0, 29.1, 22.8, 14.3. HRMS (ESI): *m*/*z* calcd. for C_42_H_42_Br_2_N_2_ [M + H]^+^ 733.1788, found 733.1756.

Synthesis of 5,11-dihexyl-N,N-bis(4-(4,4,5,5-tetramethyl-1,3,2-dioxaborolan-2-yl) phenyl)indolo[3,2,1-jk]carbazol-2-amine (9).

Compound 8 (1.50 g, 2.04 mmol), [1,1′-bis(diphenylphosphino)ferrocene] dichloropalladium(II) (0.11 g, 0.14 mmol), potassium acetate (1.01 g, 24.48 mmol), and bis(pinacolato)diboron (3.32 g, 12.24 mmol) were dissolved in 1,4-dioxane under N_2_ atmosphere, and the mixture was stirred at 90 °C overnight. After cooling to room temperature, the mixture was extracted with dichloromethane and dried over magnesium sulfate. The crude product was purified using silica gel column chromatography (dichloromethane/hexane, 3:2 *v*/*v*) and reprecipitated using ether to afford compound 9 as a pure product (1.2 g, 61%). ^1^H-NMR (CDCl_3_, 400 MHz, ppm): δ 7.85 (s, 2H), 7.81 (d, J = 1.6 Hz, 2H), 7.78 (d, J = 8.0 Hz, 2H), 7.69 (d, J = 6.8 Hz, 4H), 7.36 (dd, J = 8.4 Hz, 1.6 Hz, 2H), 7.14 (d, J = 6.8 Hz, 4H), 2.78 (t, J = 8.0 Hz, 4H), 1.72–1.68 (m, 4H), 1.39–1.30 (m, 32H), 0.90–0.87 (m, 6H). ^13^C-NMR (CDCl_3_, 100 MHz, ppm): δ 151.5, 142.6, 142.4, 137.7, 136.6, 136.0, 129.9, 127.6, 123.2, 121.8, 120.5, 119.3, 111.9, 83.7, 36.2, 32.2, 31.9, 29.1, 25.0, 22.8, 14.3. HRMS (ESI): *m*/*z* calcd. for C_54_H_66_B_2_N_2_O_4_ [M + H]^+^ 829.5281, found 829.5295.

Polymerization of PICA.

The target polymers were synthesized via Suzuki polycondensation. To a mixture of N,N-bis(4-bromophenyl)-5,11-dihexylindolo[3,2,1-jk]carbazol-2-amine (0.24 g, 0.32 mmol), 5,11-dihexyl-N,N-bis(4-(4,4,5,5-tetramethyl-1,3,2-dioxaborolan-2-yl)phenyl)indolo[3,2,1-jk]carbazol-2-amine (0.27 g, 0.32 mmol), tetrakis(triphenylphosphine)palladium (0) (0.03 g, 0.02 mmol), anhydrous toluene (8 mL) and 2 M K_2_CO_3_ solution (8 mL) were added under Ar atmosphere. The solution was stirred at 110 °C for 36 h. To terminate the polymerization reaction, a small amount of bromobenzene was added. After 1 h, a small amount of phenylboronic acid was added, and the reaction mixture was stirred for an additional hour. The solution was then poured into a flask containing 200 mL of methanol. The crude polymer was purified by Soxhlet extraction using acetone, methanol, and hexane. The resultant polymer was collected, dissolved in chloroform, and reprecipitated in methanol to obtain the pure product (107.2 mg, 29%).

### 2.3. Measurements

^1^H and ^13^C NMR spectra were recorded on a Bruker Varian and Ascend 400 MHz spectrometer in CDCl_3_. UV–Vis absorption spectra were recorded on a Shimadzu UV-3600 spectrophotometer, and photoluminescence spectra were measured on a Hitachi F-7000 spectrophotometer. Cyclic voltammetry (CV) measurements were carried out with a CHI 600D system in acetonitrile solution containing 0.1 M tetrabutylammonium tetrafluoroborate (TBABF_4_) as the supporting electrolyte, Ag/AgNO_3_ as the reference electrode, a platinum wire as the counter electrode, and a platinum working electrode. The current density–voltage–luminance (I–V–L) characteristics were measured, and the electroluminescence (EL) spectra of the phosphorescent OLEDs were recorded using a Keithley 2400 source measurement unit and a CS-1000 spectrophotometer. A Keithley 2400 source measurement unit and a CS-1000 spectroradiometer were used to evaluate the device performance. The molecular weight was measured by gel permeation chromatography (GPC) with polystyrene standard and THF as the eluent using an Agilent 1260 Series instrument. The surface morphologies were measured by atomic force microscopy (AFM; Park Systems NX-20) in the non-contact scanning mode.

### 2.4. Fabrication of OLED Devices

We fabricated red and green OLED devices. The red devices had the following two structures:

Device (A): ITO/PEDOT:PSS (30 nm)/PICA:FPA (3 wt%)/TCTA:TPBi:Ir(mphmq)_2_tmd (50:50:3 wt%, 30 nm)/TPBi (33.5 nm)/LiF (1 nm)/Al (100 nm)

Device (B): ITO/PEDOT:PSS (30 nm) Poly-TPD:FPA (3 wt%)/TCTA:TPBi:Ir(mphmq)_2_tmd (50:50:3 wt%, 30 nm)/TPBi (28 nm)/Li (1 nm)/Al (100 nm).

The indium tin oxide (ITO) substrate was cleaned by sonication in acetone and isopropyl alcohol, followed by ultraviolet/ozone treatment for 20 min. A hole-injection layer (HIL) consisting of poly(3,4-ethylenedioxythiophene) doped with poly(styrene sulfonic acid) (PEDOT:PSS, AI4083) was spin-coated onto the ITO substrate at 2000 rpm for 40 s. An HTL of poly-TPD:FPA (3 wt%) or PICA:FPA (3 wt%) was deposited onto the PEDOT:PSS layer. The coated HTL was irradiated with UV light (254 nm, 80 mJ/cm^2^) for 5 s to form a crosslinked network. The EML of TCTA:TPBi:Ir(mphmq)_2_tmd (50:50:3 wt%) was spin-coated and then annealed at 110 °C for 30 min to remove the residual solvent. The electron transport layer (ETL) of TPBi was deposited onto the EML. Finally, a layer of lithium fluoride (LiF) as the electron-injecting layer (EIL) and a layer of aluminum (Al) as the cathode were deposited by thermal evaporation on top of the film through a mask under high vacuum (below 1.0 × 10^−5^ Torr, 2.7 mPa).

Green OLED devices with the following structure were fabricated through spin-coating and vacuum deposition: ITO/PEDOT:PSS (30 nm)/PICA:FPA (3 wt%) or poly-TPD:FPA (3 wt%)/CBP:PVK:Ir(mppy)_3_ (50:50:7 wt%, 35 nm)/TPBi (40 nm)/LiF (1 nm)/Al (100 nm). The fabrication procedure was identical to that used for the red OLEDs.

## 3. Results and Discussion

### 3.1. Synthesis and Characterization of PICA

Detailed synthetic procedures for the monomers and polymers were described above in the Experimental section. The hydroxyl groups on the side chains of compound 5 were successfully reduced using triethylsilane and trifluoroacetic acid to obtain compound 6. To obtain compound 7, diphenylamine was introduced at the 2-position of indolo[3,2,1-jk]carbazole using a Buchwald–Hartwig cross-coupling reaction, followed by bromination and borylation to obtain compounds 8 and 9, respectively.

PICA was successfully polymerized through the Suzuki polycondensation of compounds 8 and 9, and the resulting polymer was soluble in organic solvents such as chloroform, toluene, and chlorobenzene. The molecular weight of PICA was measured using GPC with THF as the eluent. The number-average molecular weight (M_n_) and polydispersity index (PDI) of PICA were 8.16 kg/mol and 1.48, respectively. As a known HTL polymer, poly(4-butylphenyl-diphenylamine) (poly-TPD) with similar molecular weight was also prepared using the same Suzuki polycondensation as a reference material for comparing the hole-transporting properties. The M_n_ and PDI values of the synthesized poly-TPD were 6.92 kg/mol and 1.22, respectively. Thermal stability, optical properties, and HOMO-LUMO energy levels were measured to confirm whether PICA is suitable for application as a hole-transporting layer (HTL) of OLEDs.

The thermal properties of PICA were investigated using thermogravimetric analysis (TGA) under a nitrogen atmosphere. The 5% weight loss temperature (T_d_) of PICA was 360 °C, showing that the polymer has good thermal stability and is suitable for application in OLED devices. This temperature is comparable to that of poly-TPD (356 °C, Figure S25 in Electronic Supplementary Information (ESI†)).

**Figure 1:**
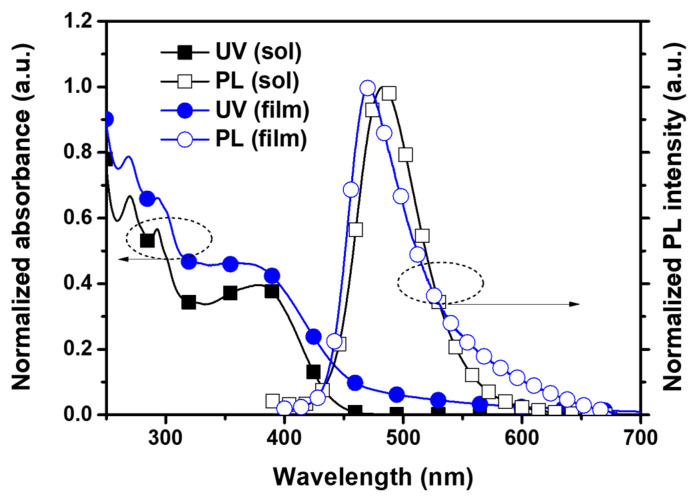
UV–Vis absorption and PL spectra of PICA in solution and film states.

The UV–Vis absorption and photoluminescence (PL) spectra of PICA in solution (1 × 10^−5^ M in chloroform) and thin-film states were measured to investigate the optical properties. PICA showed an absorption maximum at 293 nm in both states (Figure 1, Table 1). The PL maximum of PICA in the solution and film states was at 483 and 471 nm, respectively. The triplet energy of PICA was measured to be −2.33 eV from the low-temperature PL maximum, and this value is slightly lower than that of poly-TPD (−2.31 eV, Figure S26, ESI†).

**Figure 2:**
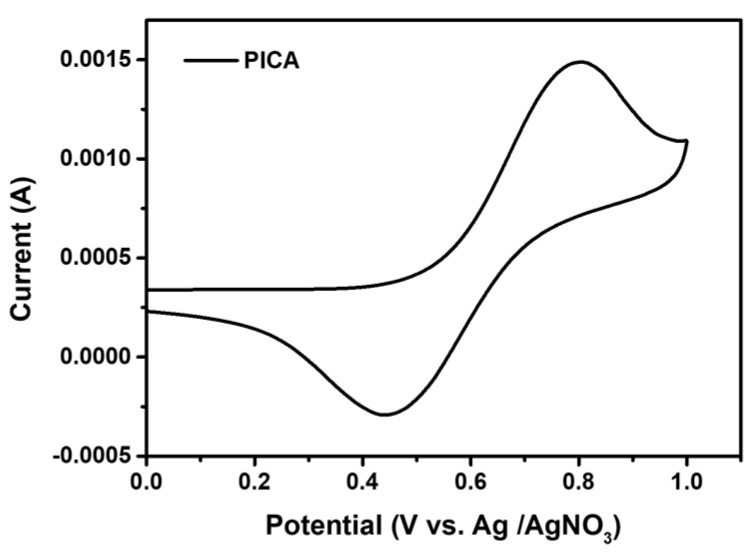
Cyclic voltammogram of PICA.

The electrochemical behavior of PICA was studied using CV in anhydrous acetonitrile (CH_3_CN) solution with 0.1 M tetrabutylammonium tetrafluoroborate as a supporting electrolyte (Figure 2). The HOMO energy level of PICA was determined by measuring the oxidation onset potential (E_ox_) of the PICA film coated on the Pt working electrode. This energy level was estimated to be −5.25 eV, slightly higher than that of poly-TPD (−5.30 eV). The lowest unoccupied molecular orbital (LUMO) energy level of PICA was estimated to be −2.46 eV using a combination of the abovementioned HOMO energy level and the energy bandgap (E_gap_) obtained from the absorption edge.

**Figure 3:**
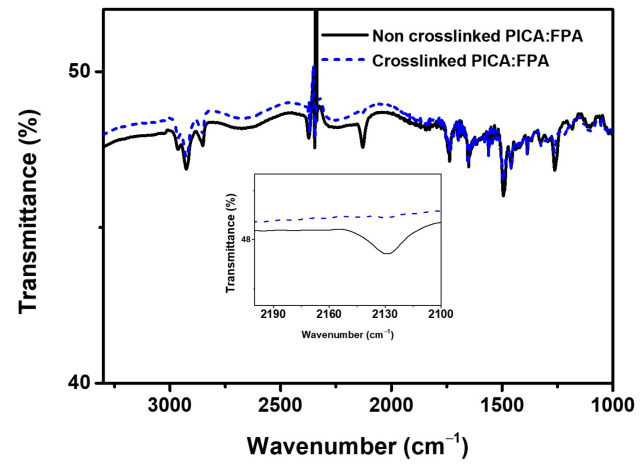
FT-IR spectra of PICA:FPA (3 wt%) films before and after crosslinking, inset: enlarged spectra.

FT-IR spectroscopy was used to confirm photo-crosslinking in the blend of PICA and FPA after UV irradiation. The blended solution of PICA:FPA was coated onto a KBr pellet, and the pellet was irradiated by 254 nm UV light. The stretching peak intensity of the terminal azide (N=N=N) in FPA at 2129 cm^−1^ decreased after irradiation, which indirectly indicates that the azide was subsequently inserted into the benzylic positions in PICA to form a network (Figure 3). The successful photo-crosslinking of PICA is also supported by the solvent resistance test results in Section 3.2 below.

### 3.2. Crosslinking and Solvent Resistance Tests

**Figure 4:**
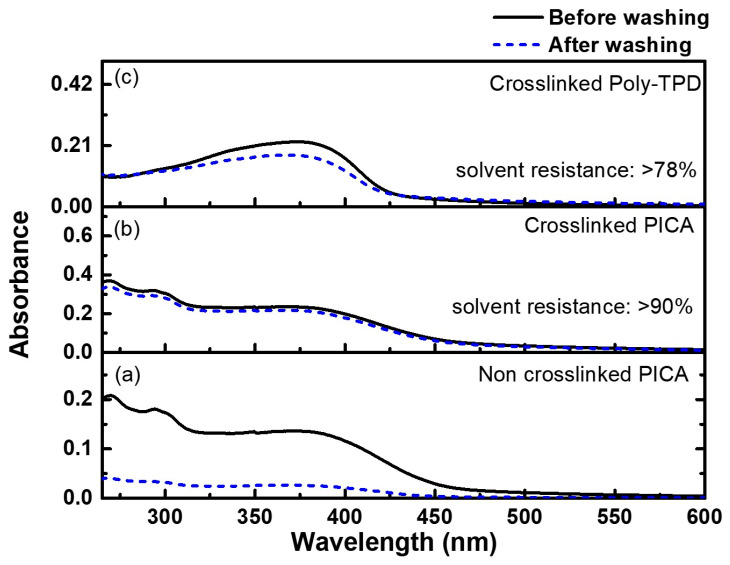
UV–Vis absorption spectra of (**a**) non-crosslinked PICA, (**b**) photo-crosslinked PICA:FPA (3 wt%), and (**c**) photo-crosslinked poly-TPD:FPA (3 wt%) films before and after rinsing with toluene.

The photo-crosslinking conditions of the **s**ynthesized PICA were optimized by performing experiments at different FPA concentrations (1, 2, 3, and 5 wt%). A mixed solution of PICA and FPA in chlorobenzene was spin-coated onto the quartz substrate. The film was then photocured by UV irradiation at 254 nm (84 mJ/cm^2^) for 5 s. After rinsing the photo-crosslinked film in toluene for 1 min, the solvent resistance was evaluated by comparing the absorbance at maximum UV–Vis absorption (294 nm for PICA and 377 nm for poly-TPD) with that before rinsing. The non-crosslinked neat PICA film showed a solvent resistance of 19.7% at 293 nm after rinsing (Figure 4a). The photo-crosslinked PICA film with 3 wt% FPA exhibited a significantly better solvent resistance of 90% (Figure 4b), whereas the poly-TPD film with 3 wt% FPA showed 78% (Figure 4c). Moreover, the absorption and PL emission spectra of the PICA films were almost identical after crosslinking (Figure S27, ESI†). It indicated that the photo-crosslinked PICA film was rarely affected by the photophysical properties even after crosslinking.

### 3.3. Hole-Only Devices Based on PICA

The hole-transporting abilities of the crosslinked PICA and poly-TPD films were evaluated by space-charge limited current (SCLC) measurements with a device structure of ITO/PEDOT:PSS (30 nm)/PICA (105 nm) or poly-TPD (95 nm)/MoO_3_(10 nm)/Al (100 nm). The carrier mobility (*μ_h_* was calculated using the following equation of Mott-Gurney law:

(1) $$\;\;J=\frac{9}{8}\varepsilon \varepsilon_{0}\frac{V^{2}}{L^{3}}\mu_{h}$$

where *ε* and *ε_0_* are the relative dielectric constant and permittivity of free space, respectively, *L* is the thickness of the organic layer, and *V* is the applied voltage. The hole mobilities of the devices based on PICA and poly-TPD films were 2.9 × 10^−5^ and 1.3 × 10^−5^ cm^2^v^–1^s^–1^, respectively (Figure S29, ESI†). The fact that PICA has more than twice the hole mobility indicates that PICA with an arylamine structure, including an indolocarbazole unit, is more advantageous for hole transport than poly-TPD.

### 3.4. Surface Morphology of PICA and Poly-TPD

**Figure 5:**
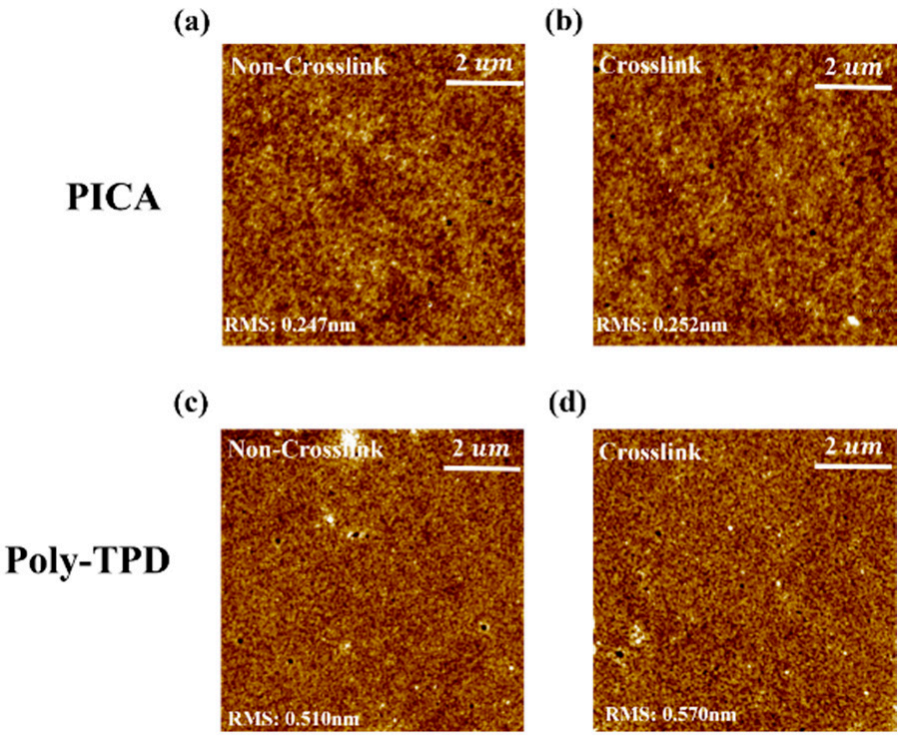
AFM images of (**a**) non-crosslinked PICA, (**c**) non-crosslinked poly-TPD, (**b**) crosslinked PICA, and (**d**) crosslinked poly-TPD films.

AFM images of the poly-TPD and PICA films were obtained to investigate changes in their surface morphology (Figure 5). The root-mean-square (RMS) roughness values of the non-crosslinked and crosslinked PICA films were 0.247 and 0.252 nm, and those of poly-TPD were 0.510 and 0.570 nm, respectively. Both the PICA and poly-TPD films showed a slight increase in RMS roughness after crosslinking, whereas PICA generated a more uniform and smoother film than poly-TPD before and after crosslinking.

### 3.5. Performances of OLEDs Based on PICA

**Figure 6:**
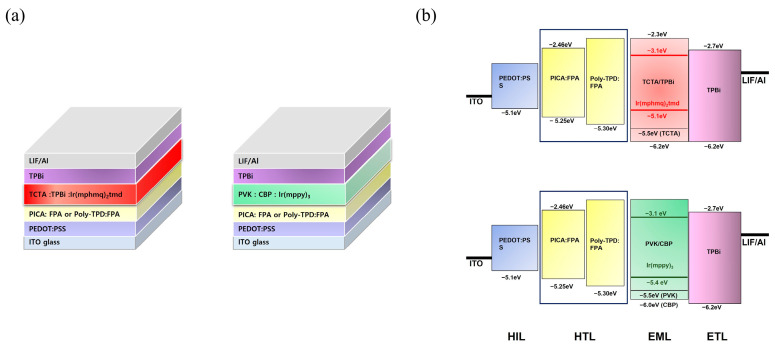
(**a**) Red and green OLED structures. (**b**) Schematic energy diagrams of the device structures.

**Figure 7:**
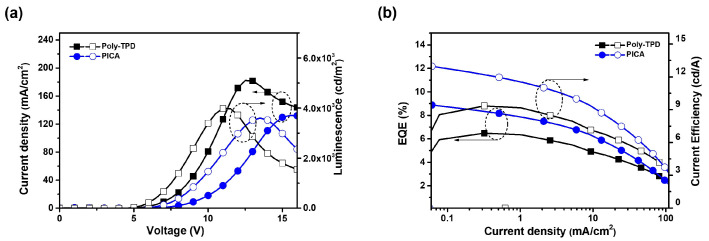
(**a**) J–V–L and (**b**) EQE–J–CE (CE = current efficiency) curves of red OLED devices using crosslinked PICA or poly-TPD as the HTL.

Red and green phosphorescent OLED devices were fabricated using crosslinked PICA to investigate the hole-transporting ability and device performance. The device structures and energy diagrams are shown in Figure 6. The device performance was compared with that of a reference device using poly-TPD. The device characteristics are listed in Table 2. Ir(mphmq)_2_(tmd) and Ir(mppy)_3_ were used as the red and green phosphorescent dopants, respectively. The red OLED device was fabricated with a structure of ITO/PEDOT:PSS (30 nm)/[PICA or poly-TPD]/TCTA:TPBi:Ir(mphmq)_2_tmd (50:50:3 wt%, 30 nm)/TPBi (33.5 nm for PICA and 28 nm for poly-TPD)/LiF (1 nm)/Al (100 nm). The turn-on voltage (V_turn-on_) and maximum luminescence (L_max_) of the red OLED devices were 5.0 V and 3600 cd/m^2^ for PICA and 4.5 V and 4000 cd/m^2^ for poly-TPD, respectively. The red OLED device based on PICA was superior to that based on poly-TPD in terms of maximum external quantum efficiency (EQE_max_, 8.88% vs. 6.47%), maximum luminous efficiency (LE_max_, 12.97 vs. 9.35 cd/A), and maximum power efficiency (PE_max_, 8.12 vs. 5.96 lm/W), as shown in Figure 7.

**Figure 8:**
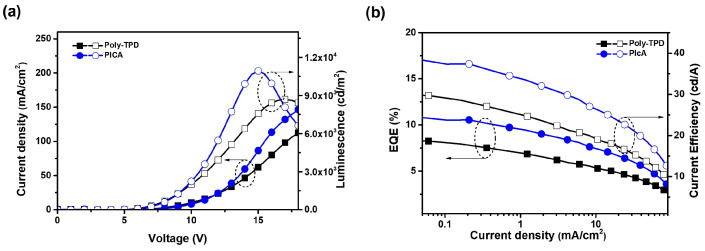
(**a**) J–V–L and (**b**) EQE–J–CE (CE = current efficiency) curves of green OLED devices using crosslinked PICA or poly-TPD as the HTL.

Green OLED devices were fabricated using the same procedure as that for the red ones with the following device structure: ITO/PEDOT:PSS (30 nm)/[PICA or poly-TPD]/CBP:PVK:Ir(mppy)_3_ (50:50:7 wt%, 35 nm)/TPBi (40 nm)/LiF (1 nm)/Al (100 nm). The CIE coordinates of the green OLEDs with crosslinked PICA and poly-TPD were (0.29, 0.64) and (0.30, 0.64), respectively. The green OLED devices showed V^turn-on^ = 4.0 V and LE_max_ = 10,900 cd/m^2^ for PICA:FPA (3 wt%), and V_turn-on_ = 4.0 V and LE_max_ = 8700 cd/m^2^ for poly-TPD:FPA (3 wt%). In the green OLED devices, PICA performed better than poly-TPD in terms of EQE_max_ (10.84% vs. 8.40%), LE_max_ (38.47 vs. 30.19 cd/A), and PE_max_ (25.06 vs. 21.07 lm/W), as shown in Figure 8. We speculate that this improved device performance of PICA compared to poly-TPD as the HTL in solution-processed OLEDs is due to the higher hole mobility and more suitable energy matching of PICA. This resulted in better hole injection from PEDOT:PSS and better charge balance, hence the enhanced device performance.

## 4. Conclusions

We successfully synthesized a new cross-linkable material, PICA, for use as HTL in solution-processed OLEDs. Red and green OLED devices using photo-crosslinked PICA (EQE/LE/PE: 8.88%/12.97/8.12 in red devices, 10.84%/38.47 cd/A/25.06 lm/W in green devices) as the HTL exhibited better device performance than reference devices using poly-TPD. The higher solvent resistance of crosslinked PICA (90%) compared to that of poly-TPD (78%) might help prevent interlayer mixing and the dissolution of the HTL when forming an upper organic layer. Moreover, it is believed that PICA shows improved device performance compared to poly-TPD as an HTL for solution-processed OLEDs due to the higher hole mobility and better energy matching with the adjacent organic layers. These results show that PICA is a good candidate for photo-crosslinkable HTL material with highly efficient hole transport and good solvent resistance for solution-processed OLEDs.

## Figures and Tables

**Table 1:** Optical and electrical properties of PICA.

Material	Abs. λ_max_ (nm)	Abs. λ_edge (_nm)	PL λ_max_ (nm)	E_g opt_ (eV)	HOMO (eV)	LUMO (eV)
PICA	270, 293	444	483	2.79	−5.25	−2.46

**Table 2:** Device performances of red and green phosphorescent OLEDs.

HTL	Device	V_turn-on_ ^a^ (V)	EQE_max_ (%)	CIE ^b^ (x,y)	L_max_ (cd/m^2^)	LE_max_ (cd/A)	PE_max_ (lm/W)	Hole Mobility (cm^2^/V·s)
Poly-TPD	Red	4.5	6.47	(0.64,0.35)	4000	9.35	5.96	1.3 × 10^−5^
	Green	4.0	8.40	(0.30,0.64)	8700	30.19	21.07	
PICA	Red	5.0	8.88	(0.64, 0.36)	3600	12.97	8.12	2.9 × 10^−5^
	Green	4.0	10.84	(0.29, 0.64)	10,900	38.47	25.06	

^a^ Measured at a luminance of 1 cd/m^2^. ^b^ Measured at a luminance of 1000 cd/m^2^.

## Data Availability

The data that support the findings of this study are available from the corresponding author upon reasonable request.

## Supplementary Materials

The following supporting information can be downloaded at: https://www.mdpi.com/article/10.3390/nano13131934/s1: Figure S1–S24: ^1^H and ^13^C NMR spectrum of compound 1~12; Figure S25: TGA data of PICA and Poly-TPD; Figure S26: Photoluminescence spectra of Poly-TPD and PICA at 77K in MeTHF solutions; Figure S27: Photoluminescence spectra of PICA with crosslinking and non-crosslinking; Figure S28: Solvent resistance of PICA with the different ratios of FPA; Figure S29: Summary of SCLC data of hole-only devices of PICA and Poly-TPD.
