# Supplementary material for: Indolocarbazole-Based Photo-Crosslinkable Hole-Transporting Layer for Efficient Solution-Processed Organic Light-Emitting Diodes
Source: Nanomaterials (Basel). 2023 Jun 25;13(13):1934. doi: 10.3390/nano13131934 (PMC10343803; doi:10.3390/nano13131934)
Supplement: Supplementary file 1 [file nanomaterials-13-01934-s001.zip › nanomaterials-2442615-supplementary.pdf]

Supproting Information

Indolocarbazole-Based Photo-Crosslinkable Hole-Transporting Layer for Efficient Solution-Processed Organic Light-Emitting Diodes

Jeong Yong Park [1], Seon Lee Kwak [1], Hea Jung Park[2,*] and Do-Hoon Hwang [1,*]

[1] Department of Chemistry and Chemistry Institute for Functional Materials, Pusan National University, Busan 46241, Republic of Korea; pjy9189@naver.com (J.Y.P.); gsun0331@naver.com (S.L.K.)

[2] Department of Biology and Chemistry, Changwon National University, Changwon 51140, Republic of Korea

* Correspondence: hjpark@changwon.ac.kr (H.J.P.); dohoonhwang@pusan.ac.kr (D.-H.H.)

Table of Contents

Section S1. ^{1}H- and ^{13}C-NMR spectra

Section S2. Thermal stability of PICA and Poly-TPD

Section S3. Photoluminescence property of PICA

Section S4. Solvent resistance test of PICA with different ratios of FPA

Section S5. SCLC data of PICA and Poly-TPD

Section S1. ^{1}H- and ^{13}C-NMR spectra

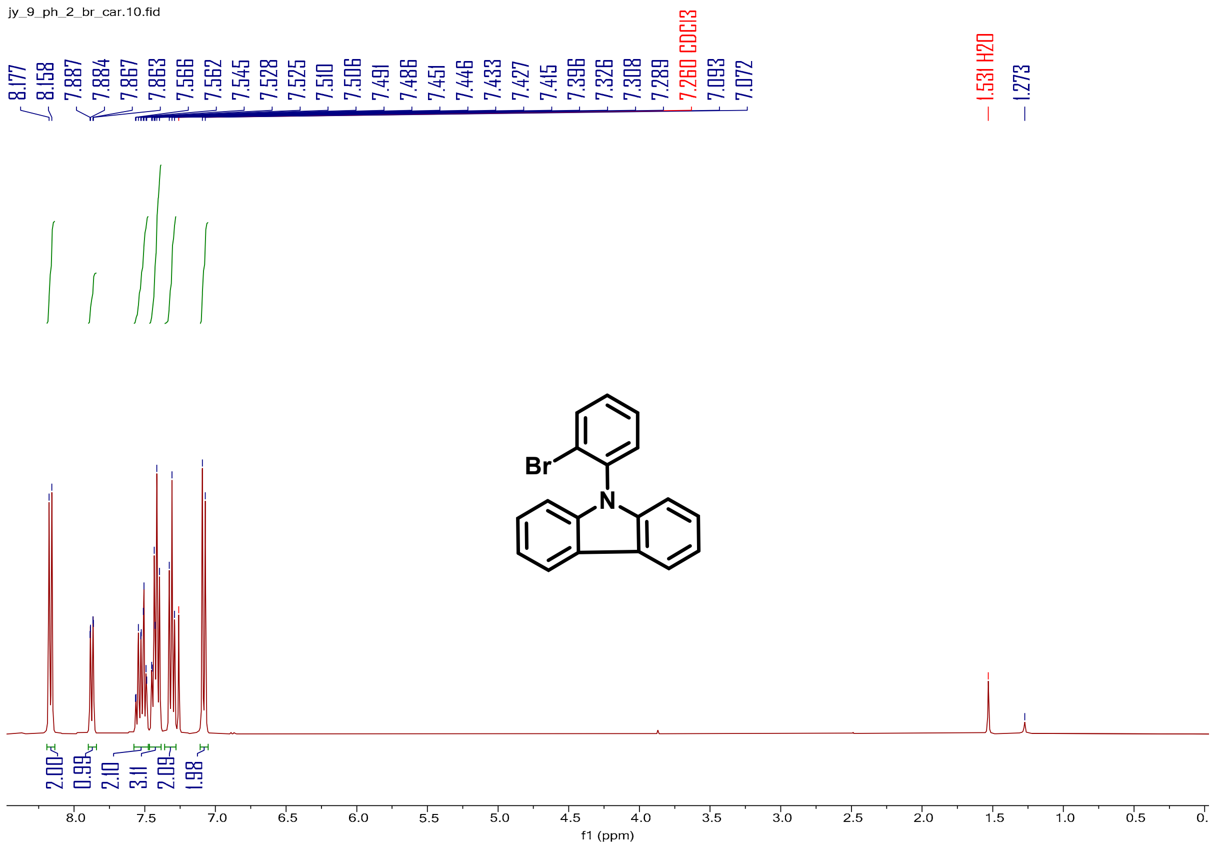

Figure S1. ^{1}H NMR spectrum of compound **1**.

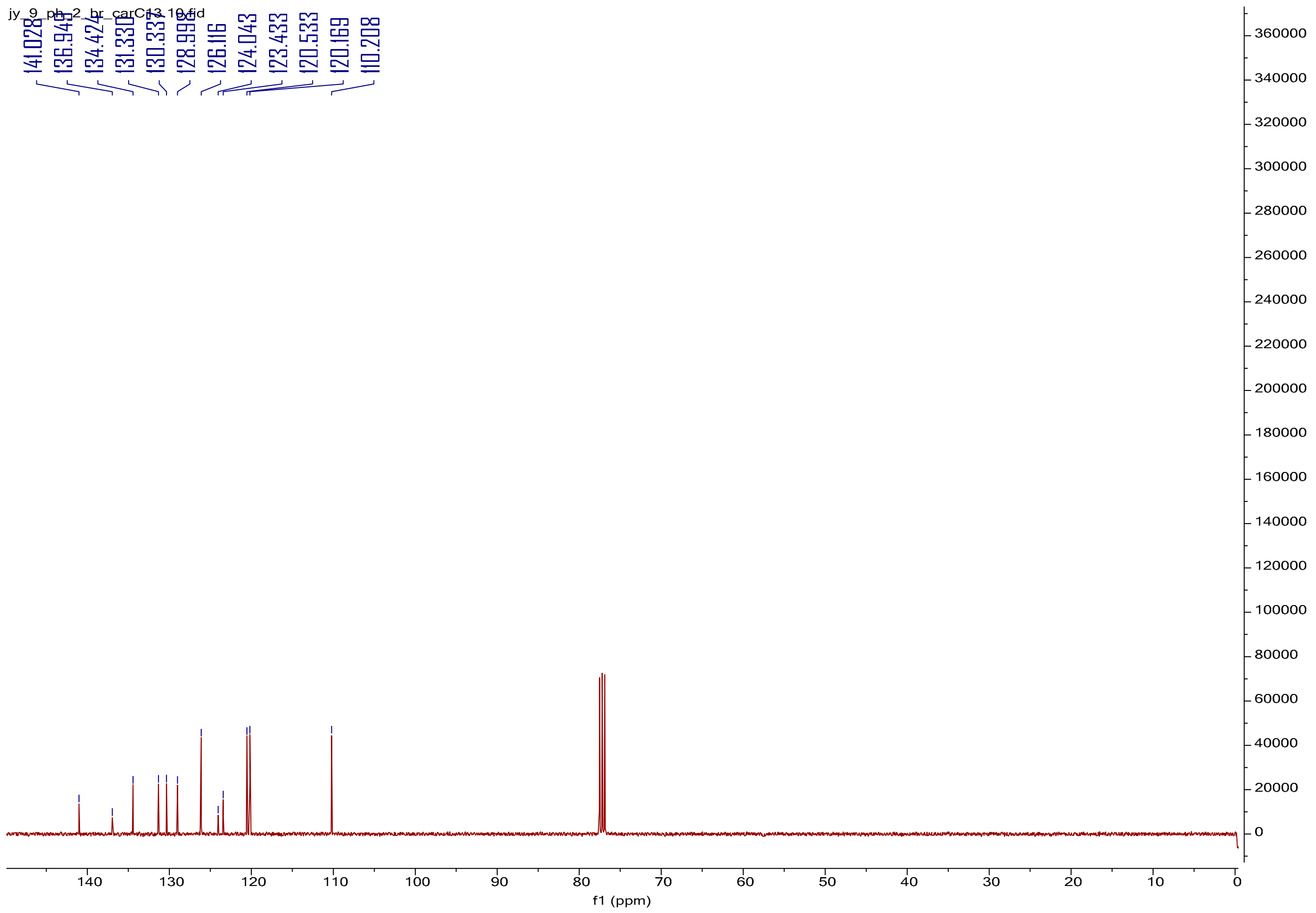

Figure S2. ^{13}C NMR spectrum of compound **1**.

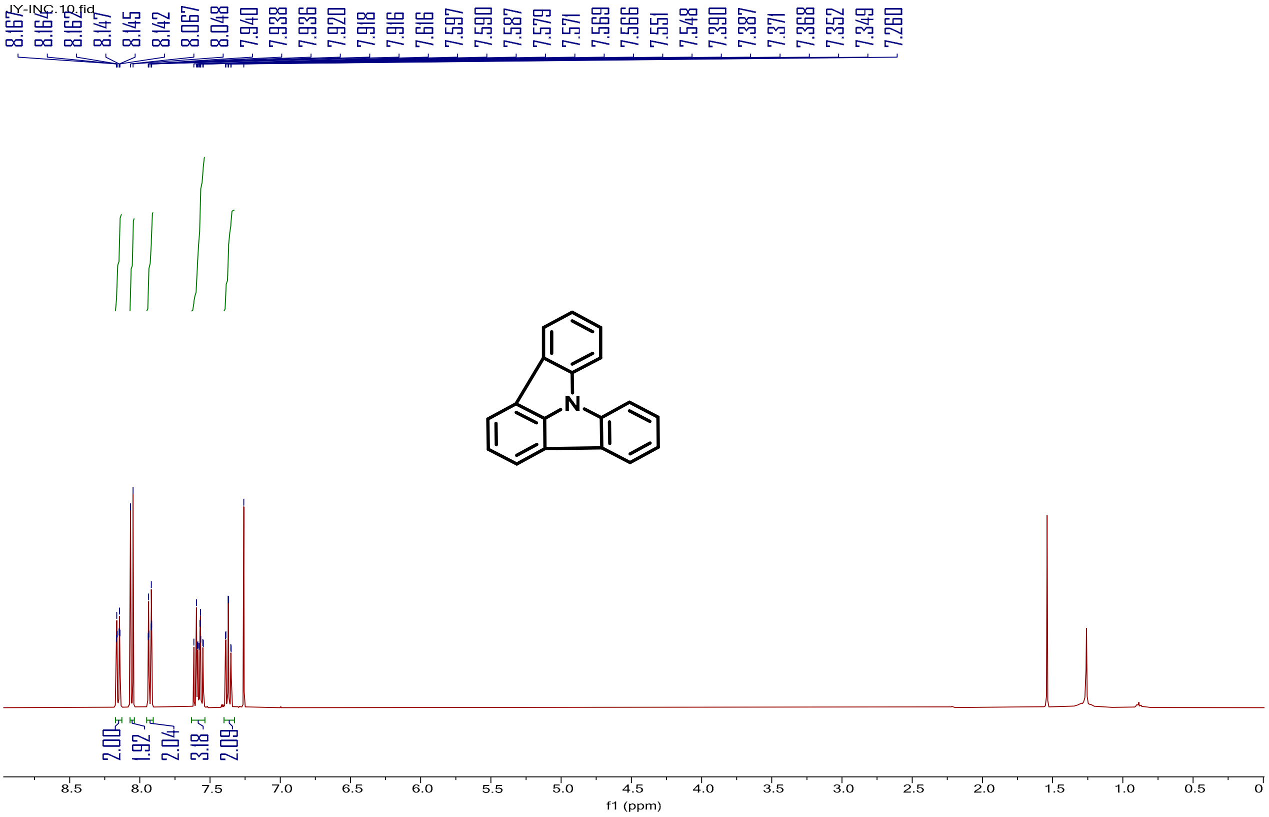

Figure S3. ^{1}H NMR spectrum of compound **2**.

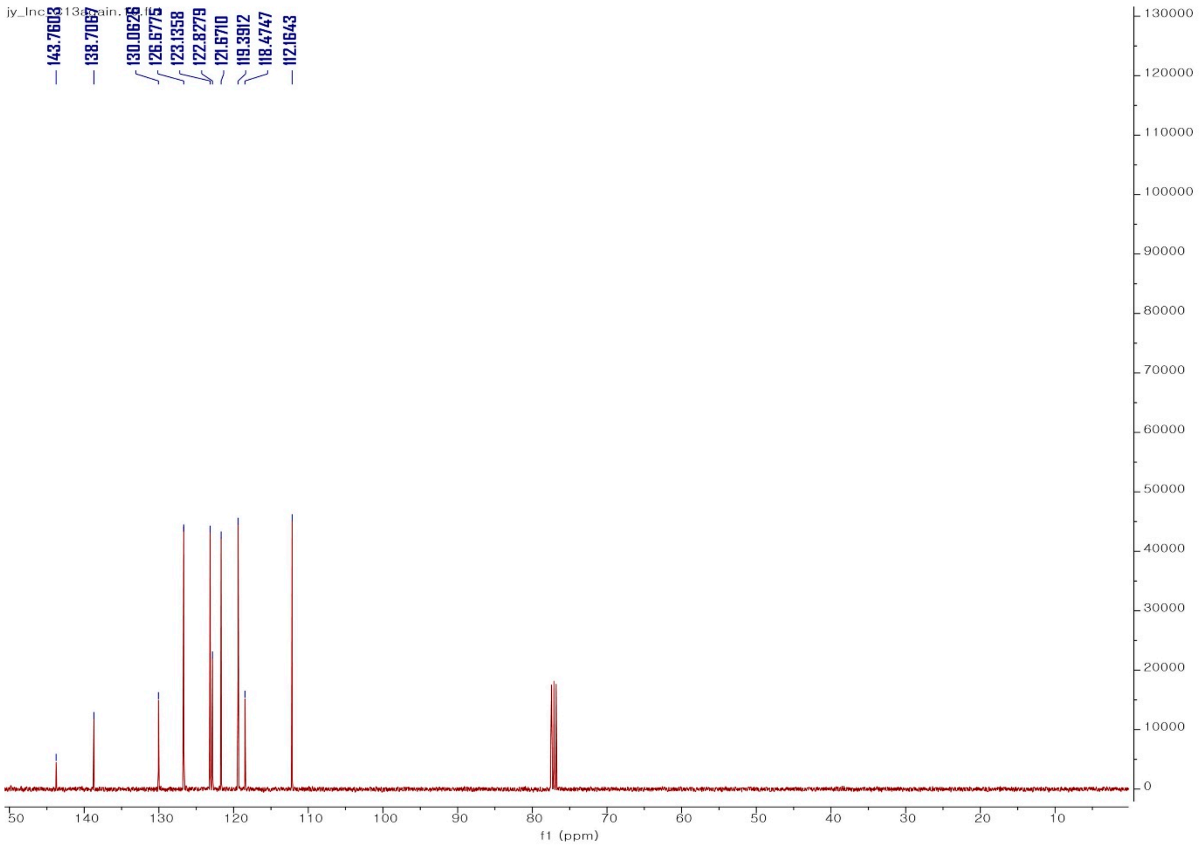

Figure S4. ^{13}C NMR spectrum of compound **2**.

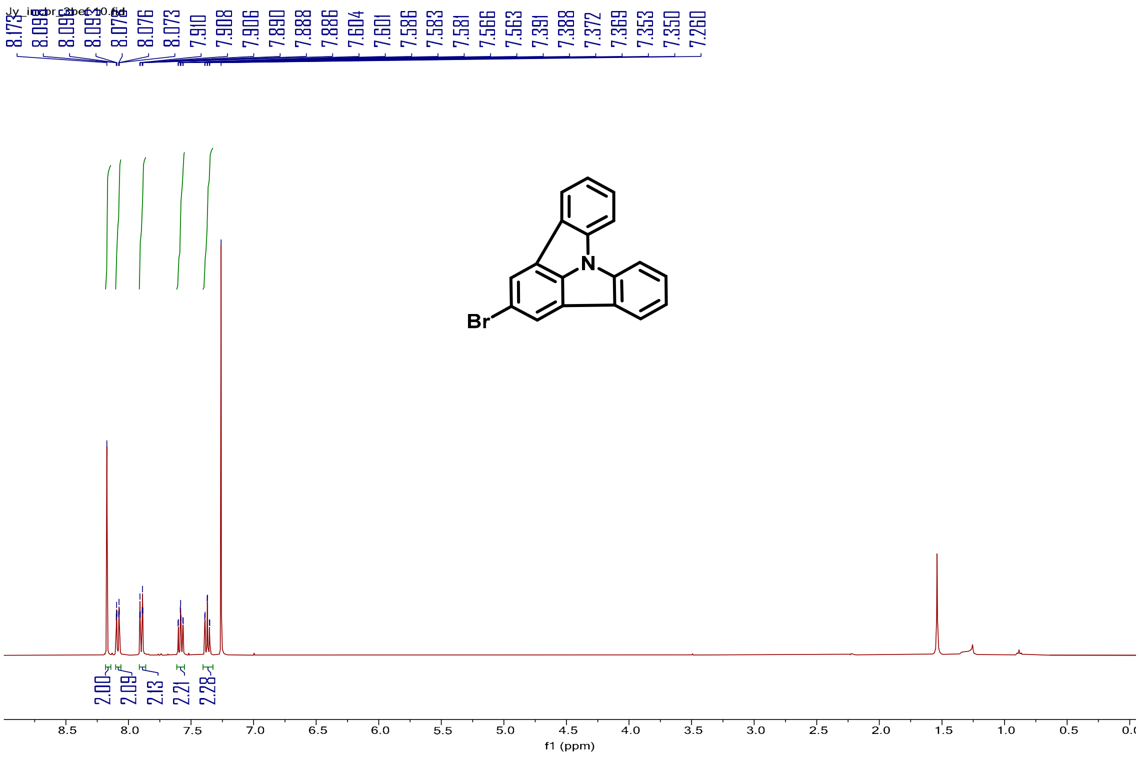

Figure S5. ^{1}H NMR spectrum of compound **3**.

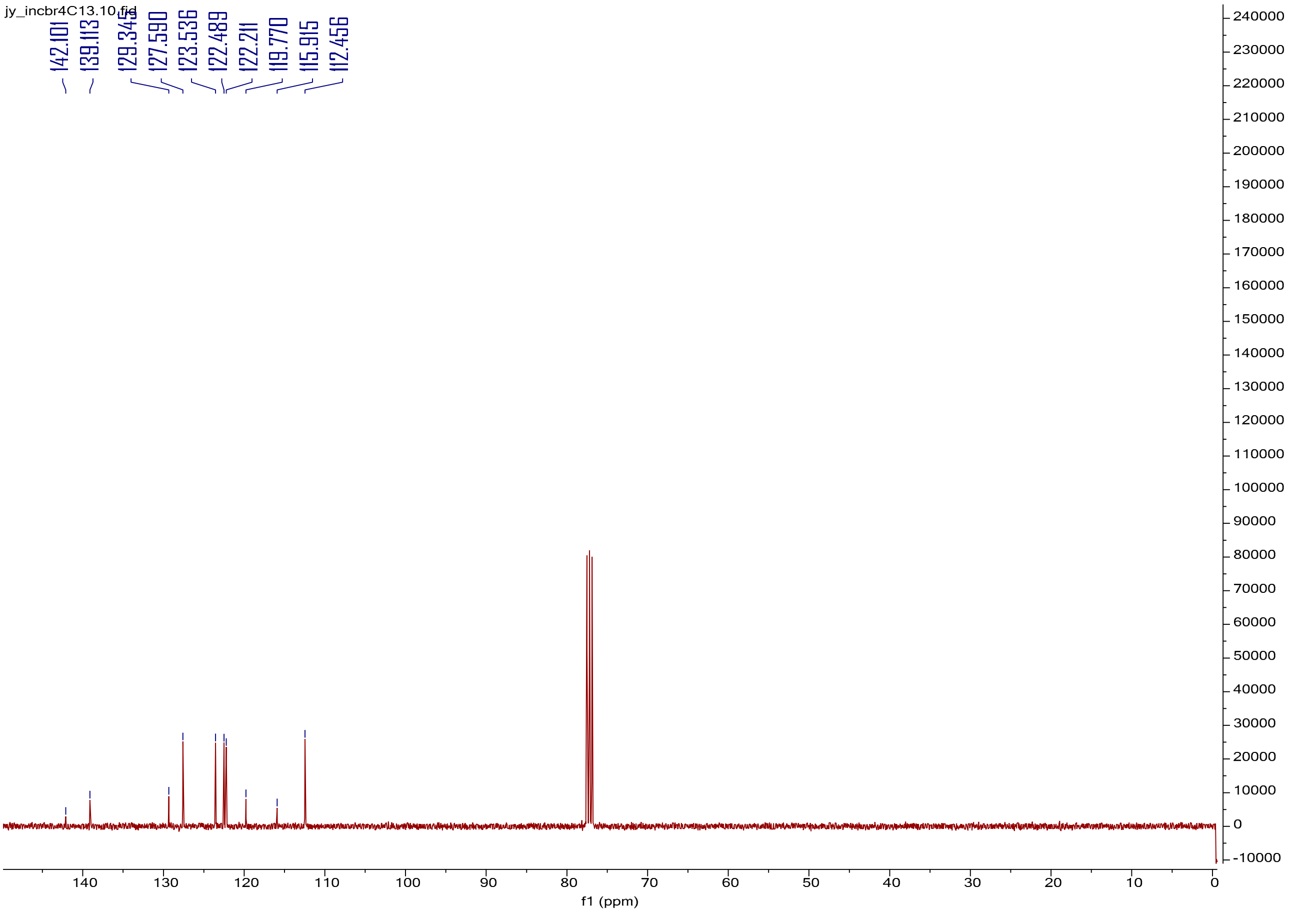

Figure S6. ^{13}C NMR spectrum of compound **3**.

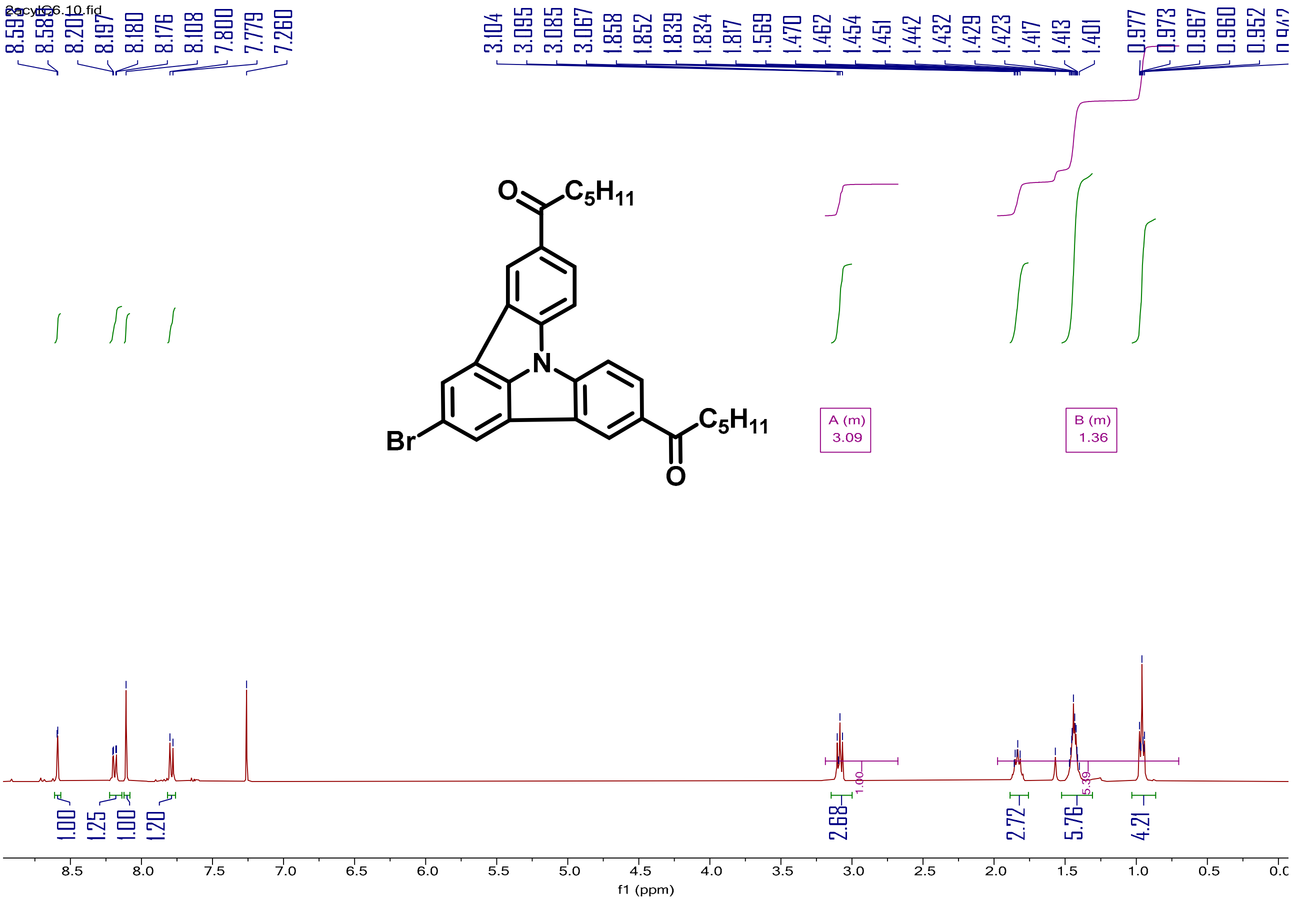

Figure S1. ^{1}H NMR spectrum of compound **4**.

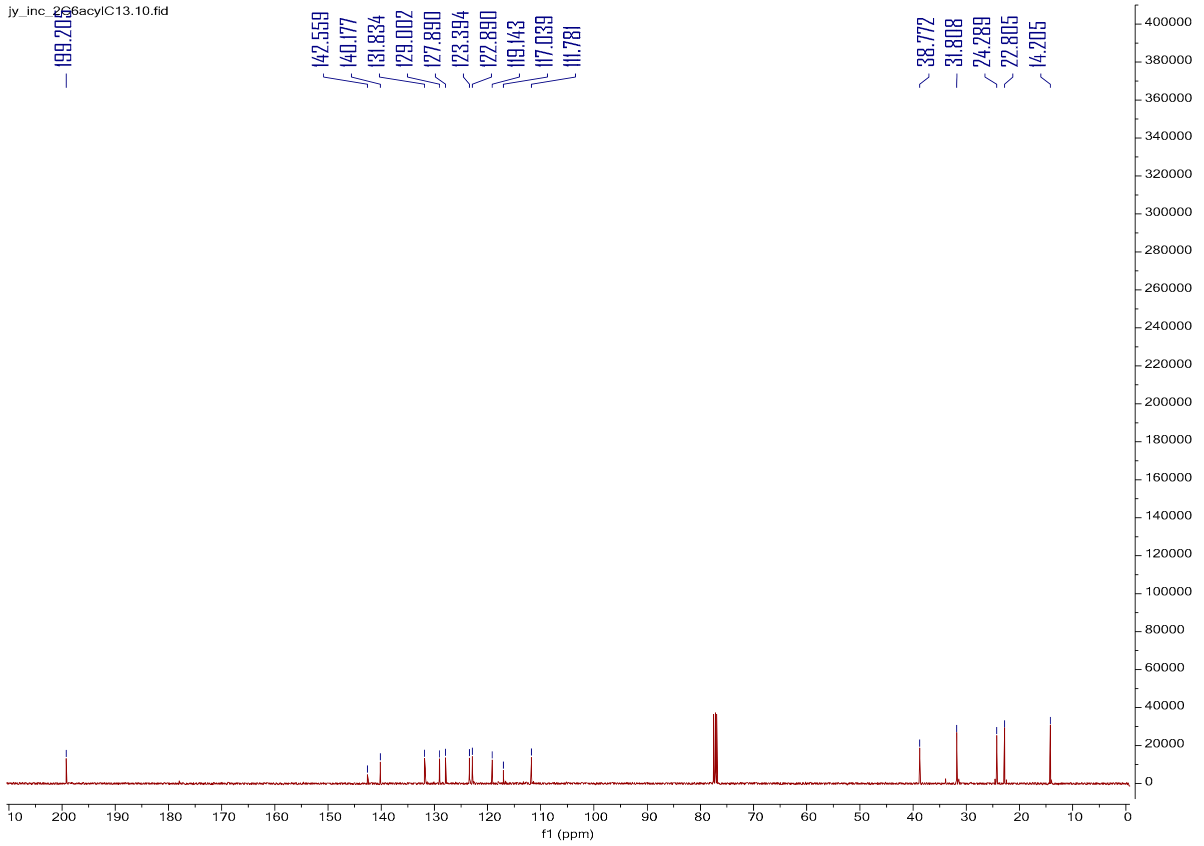

Figure S2. ^{13}C NMR spectrum of compound **4**.

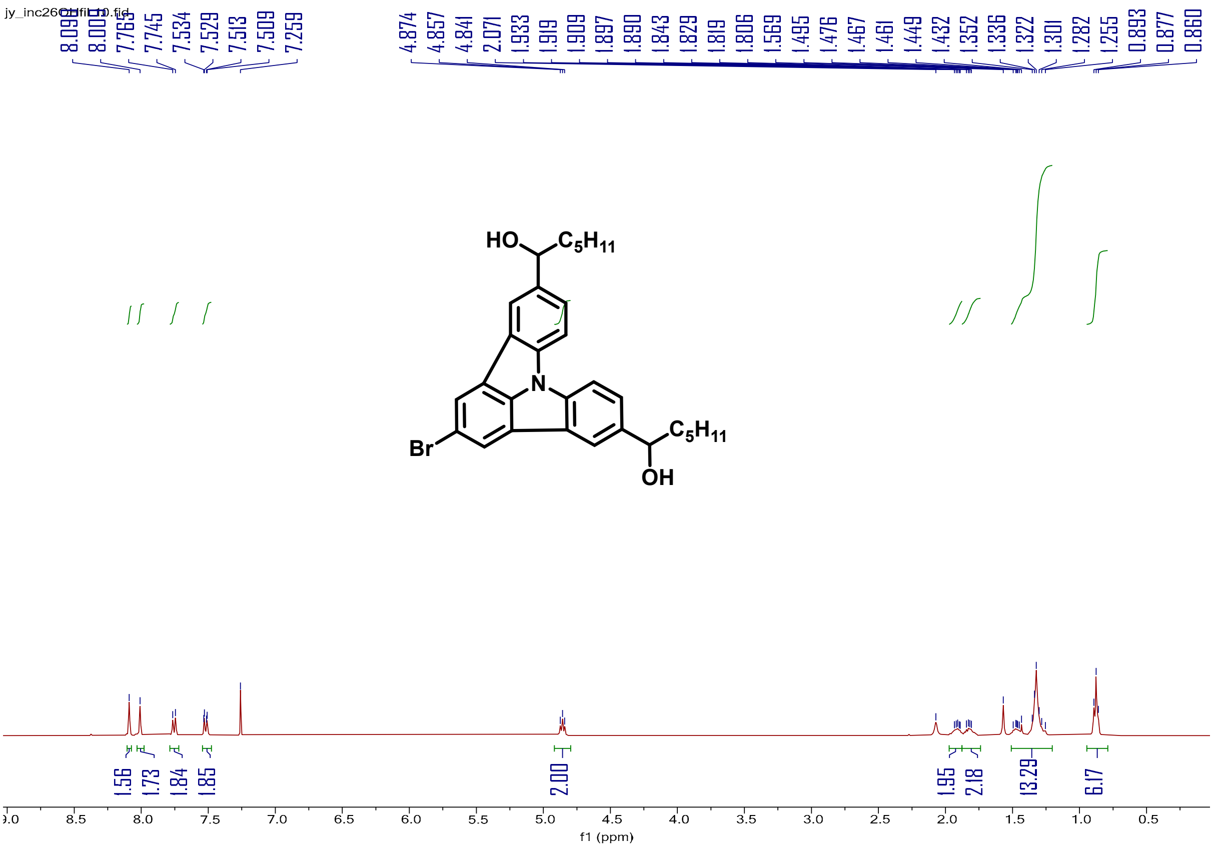

Figure S3. ^{1}H NMR spectrum of compound **5**.

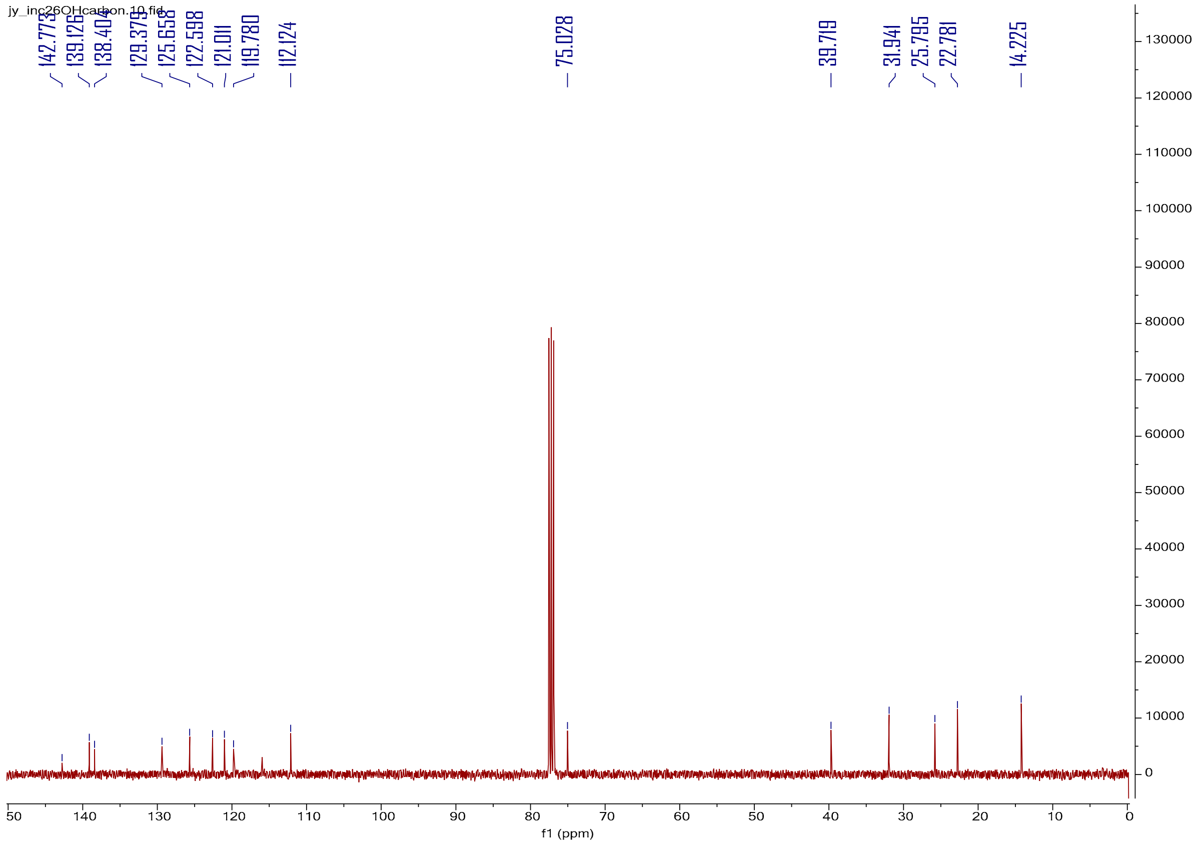

Figure S4. ^{13}C NMR spectrum of compound **5**.

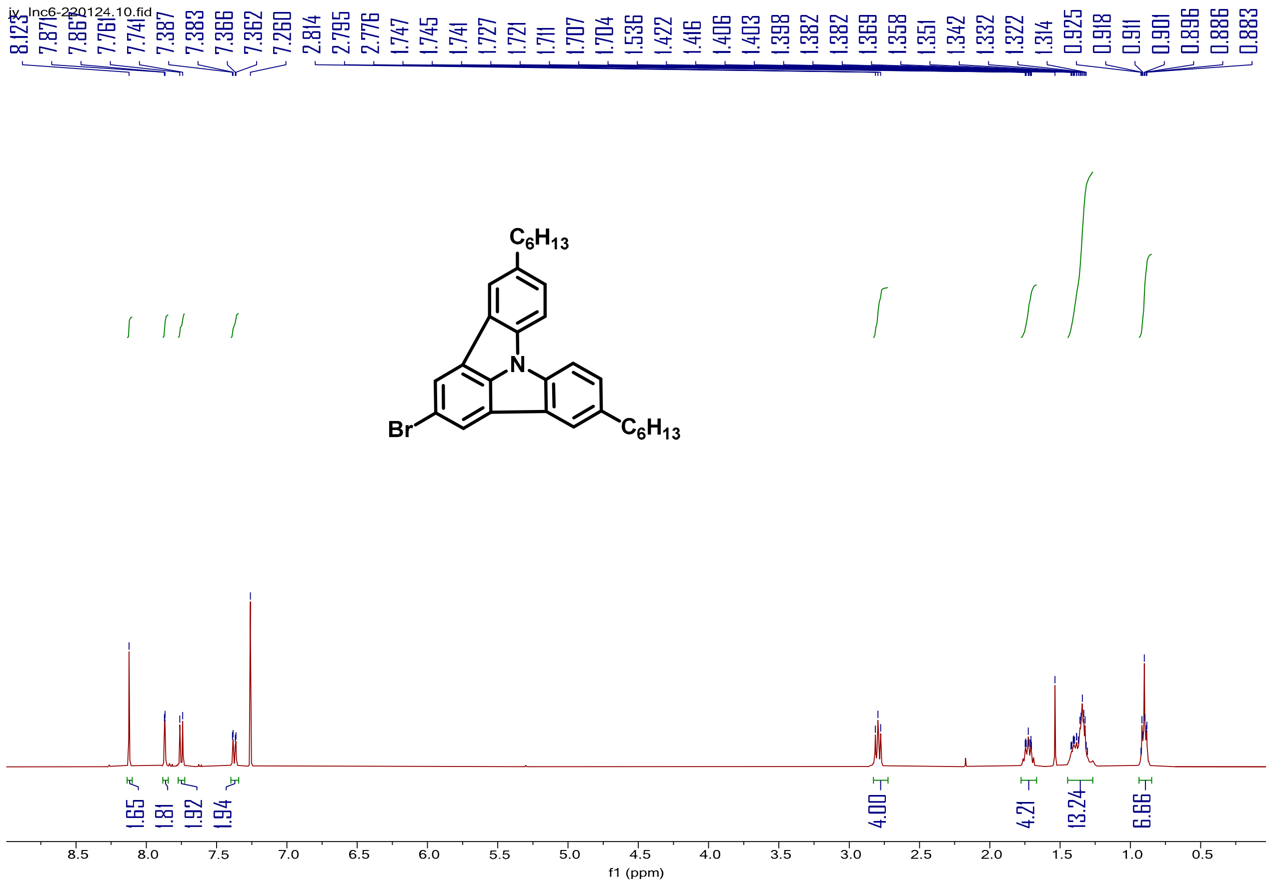

Figure S5. ^{1}H NMR spectrum of compound **6**.

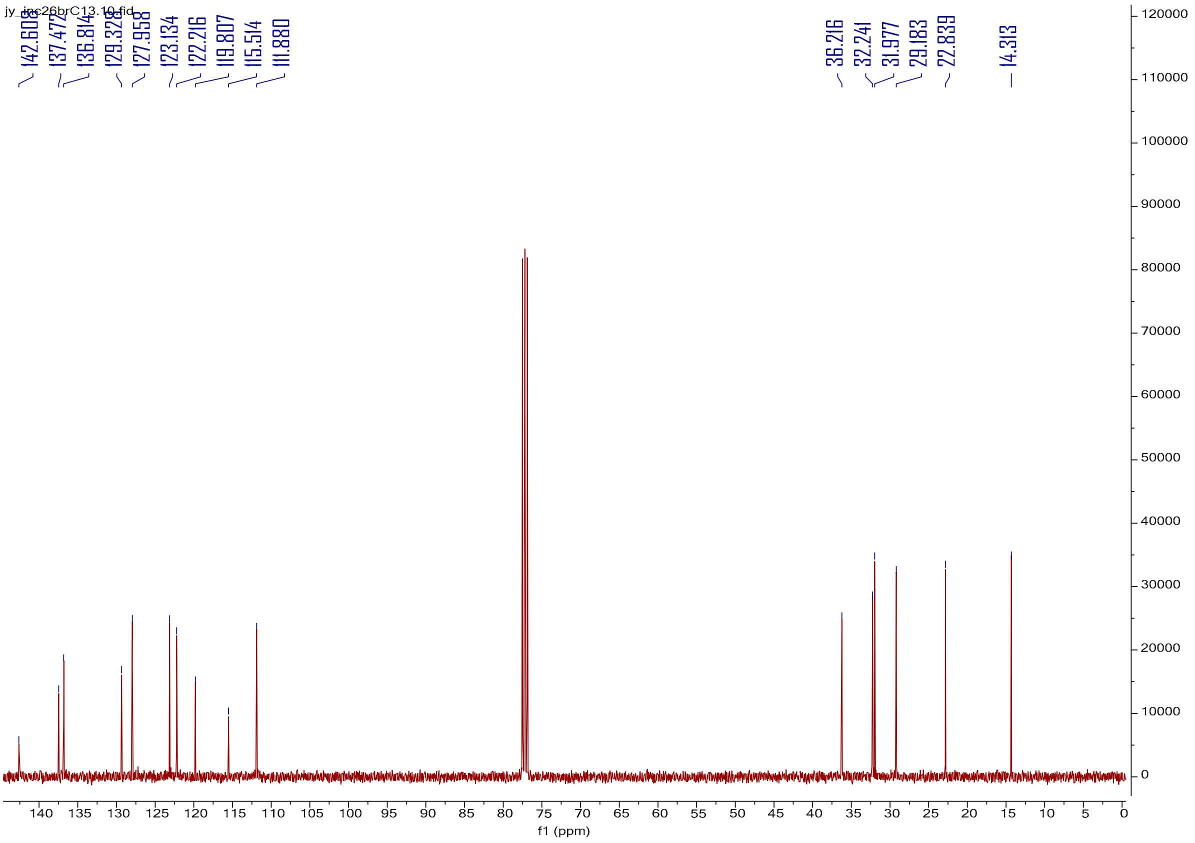

Figure S6. ^{13}C NMR spectrum of compound **6**.

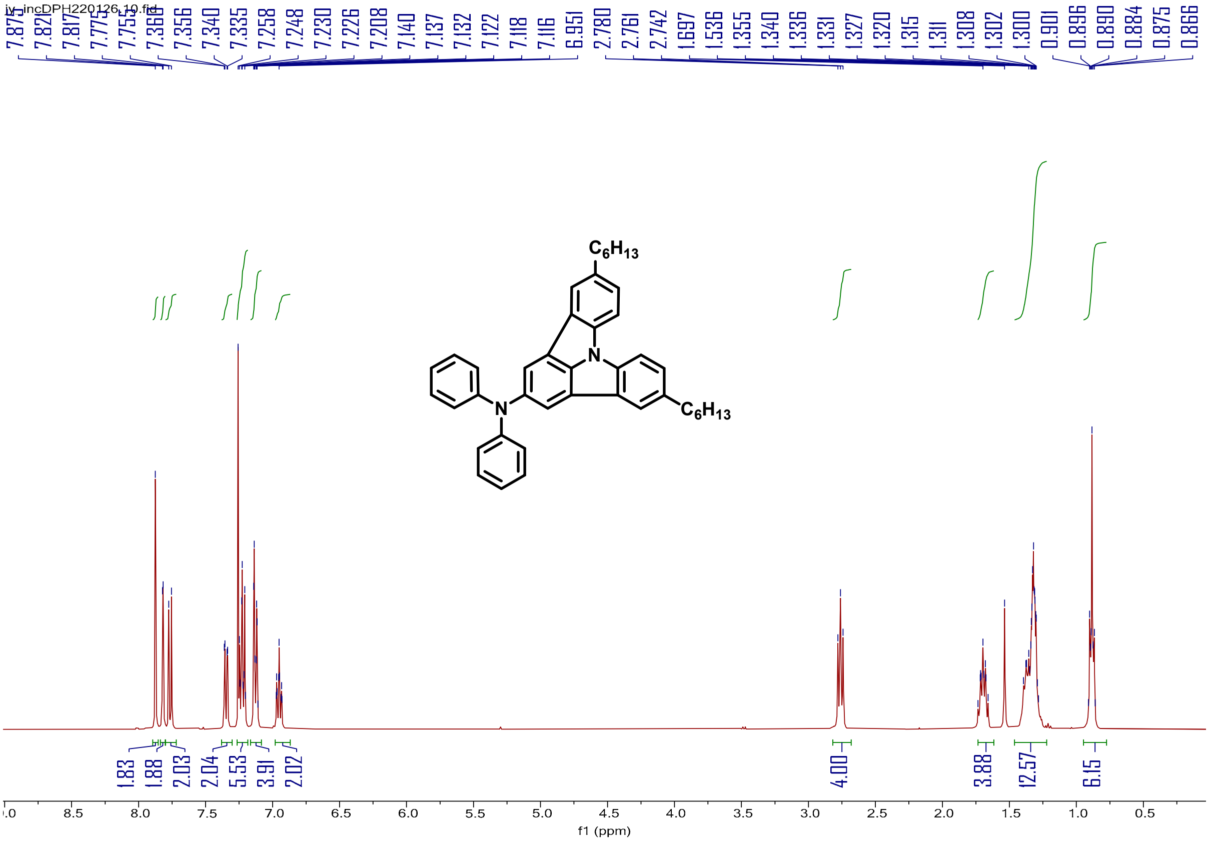

Figure S7. ^{1}H NMR spectrum of compound **7**.

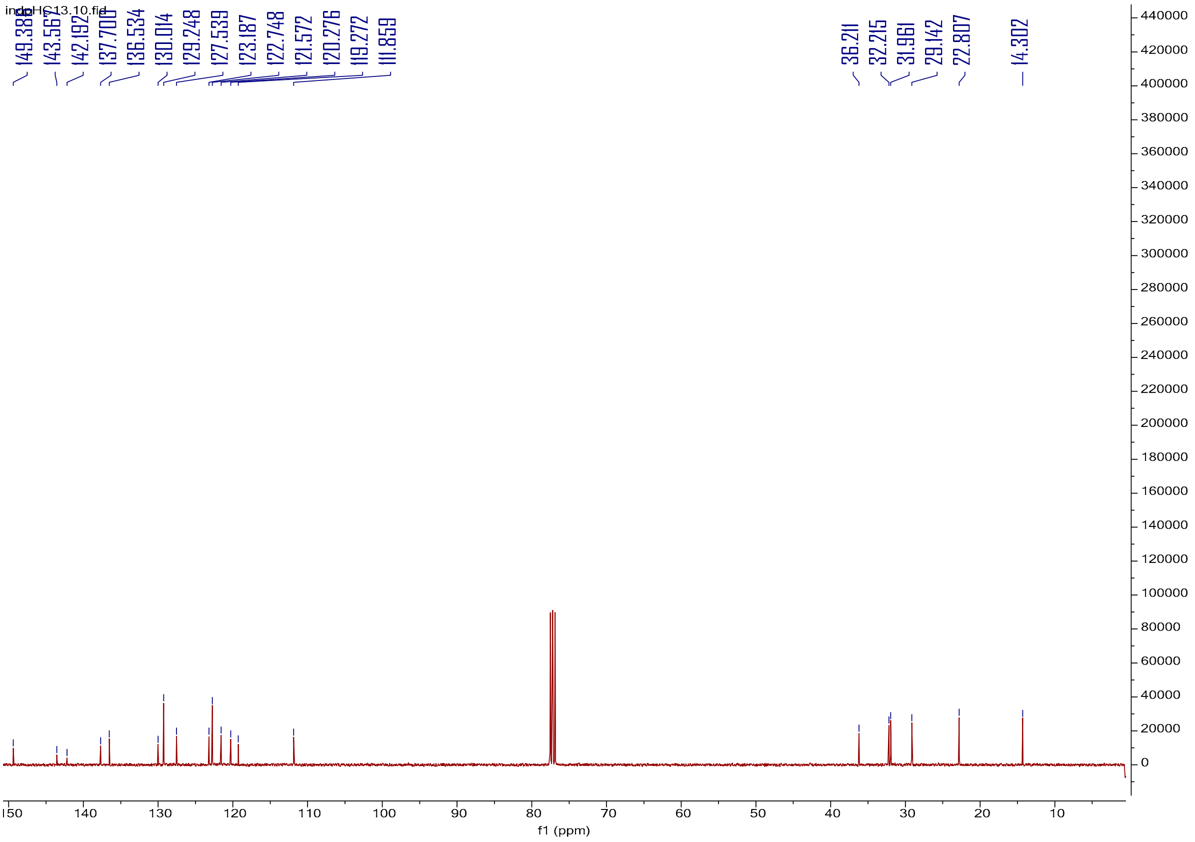

Figure S8. ^{13}C NMR spectrum of compound **7**.

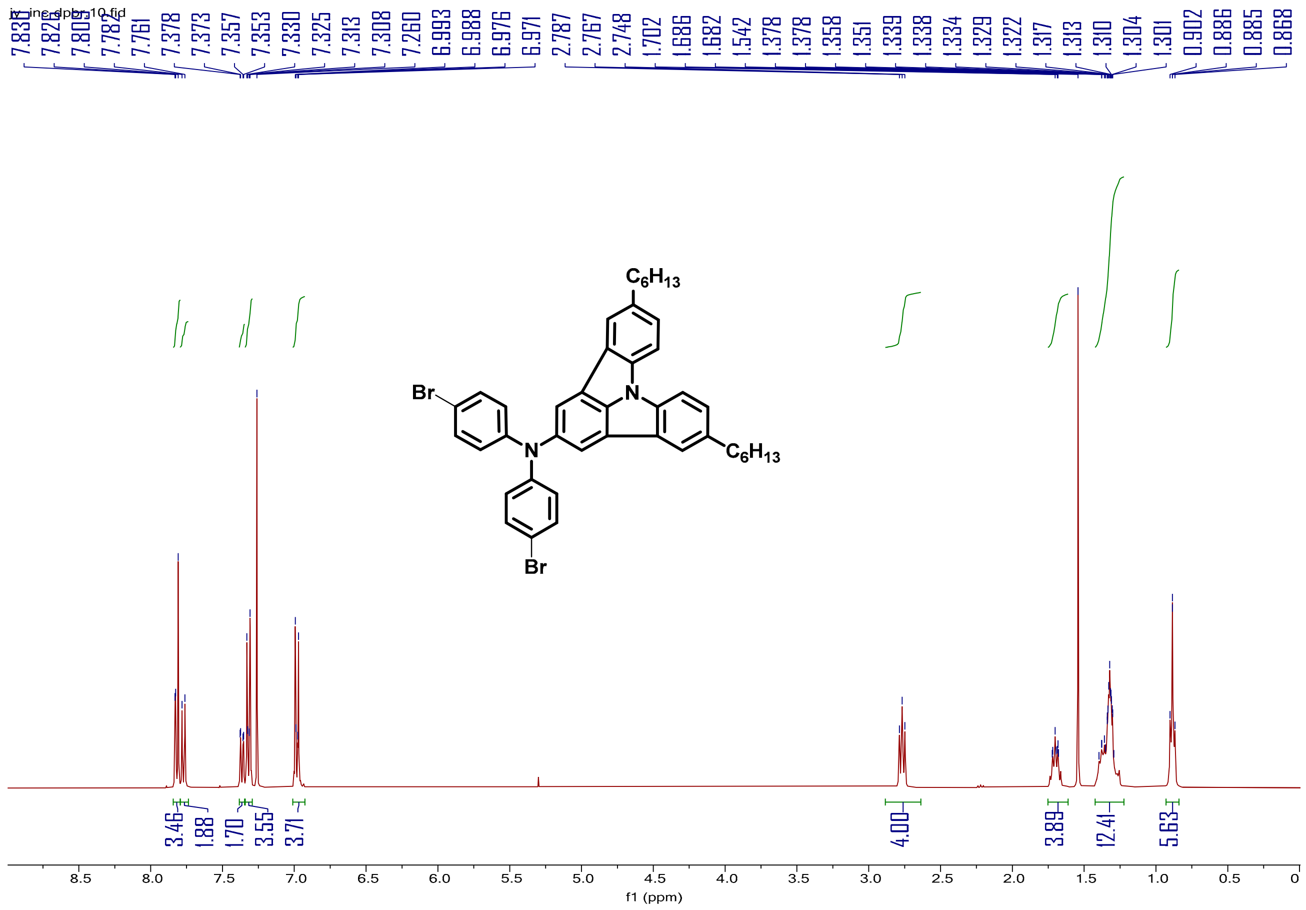

Figure S9. ^{1}H NMR spectrum of compound **8**.

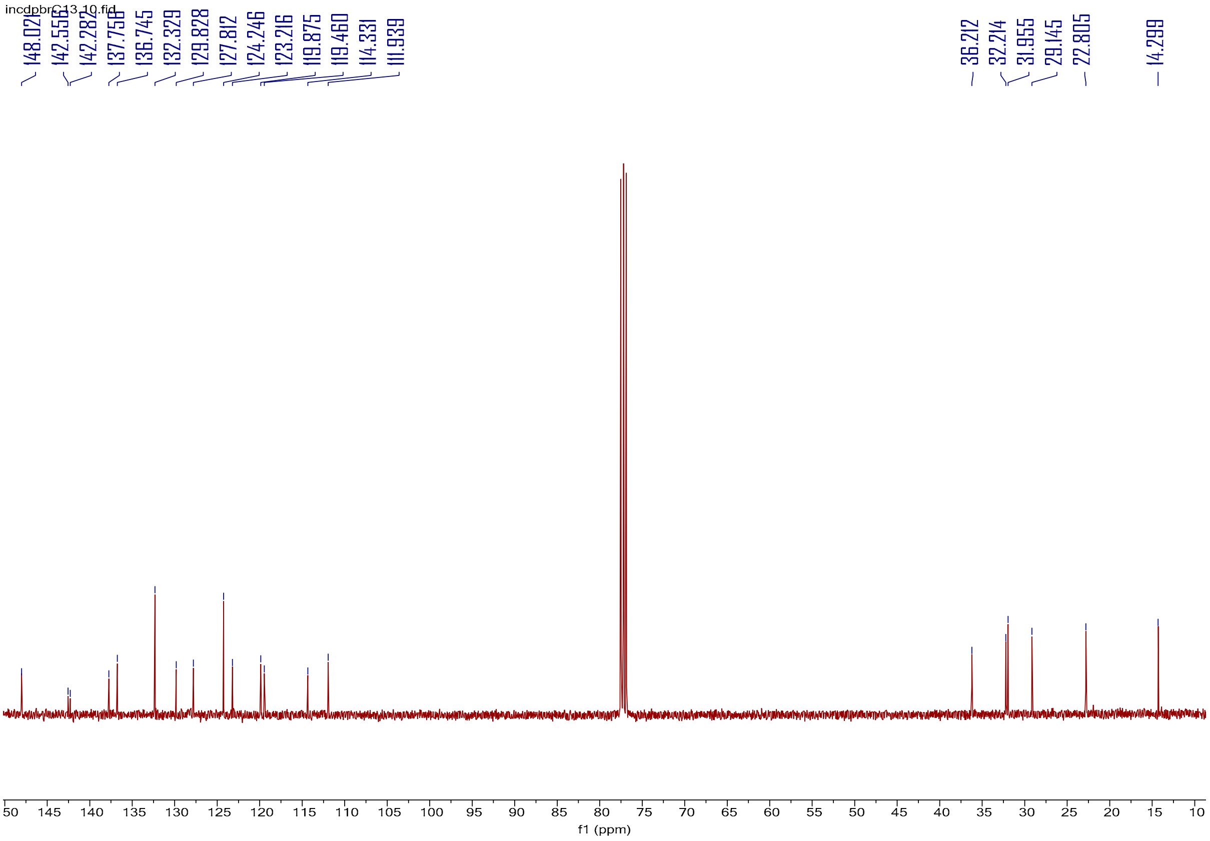

Figure S10. ^{13}C NMR spectrum of compound **8**.

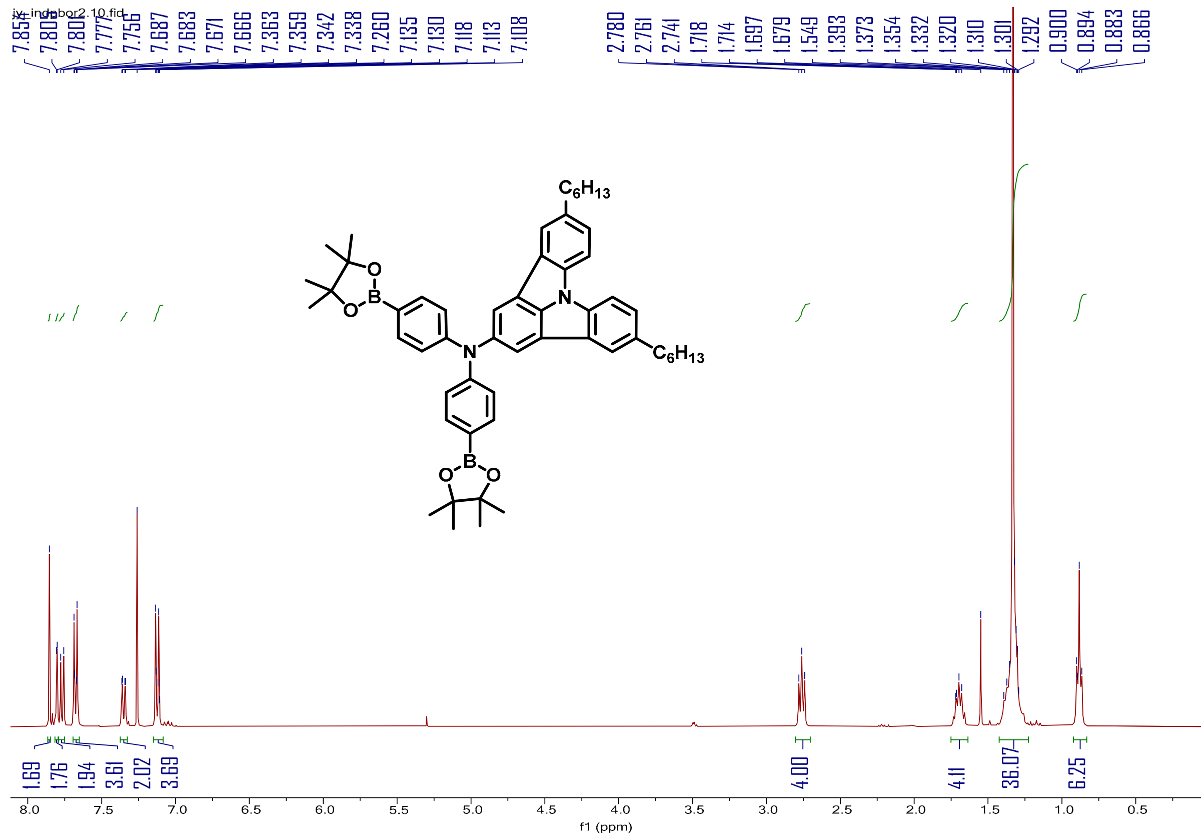

Figure S11. ^{1}H NMR spectrum of compound **9**.

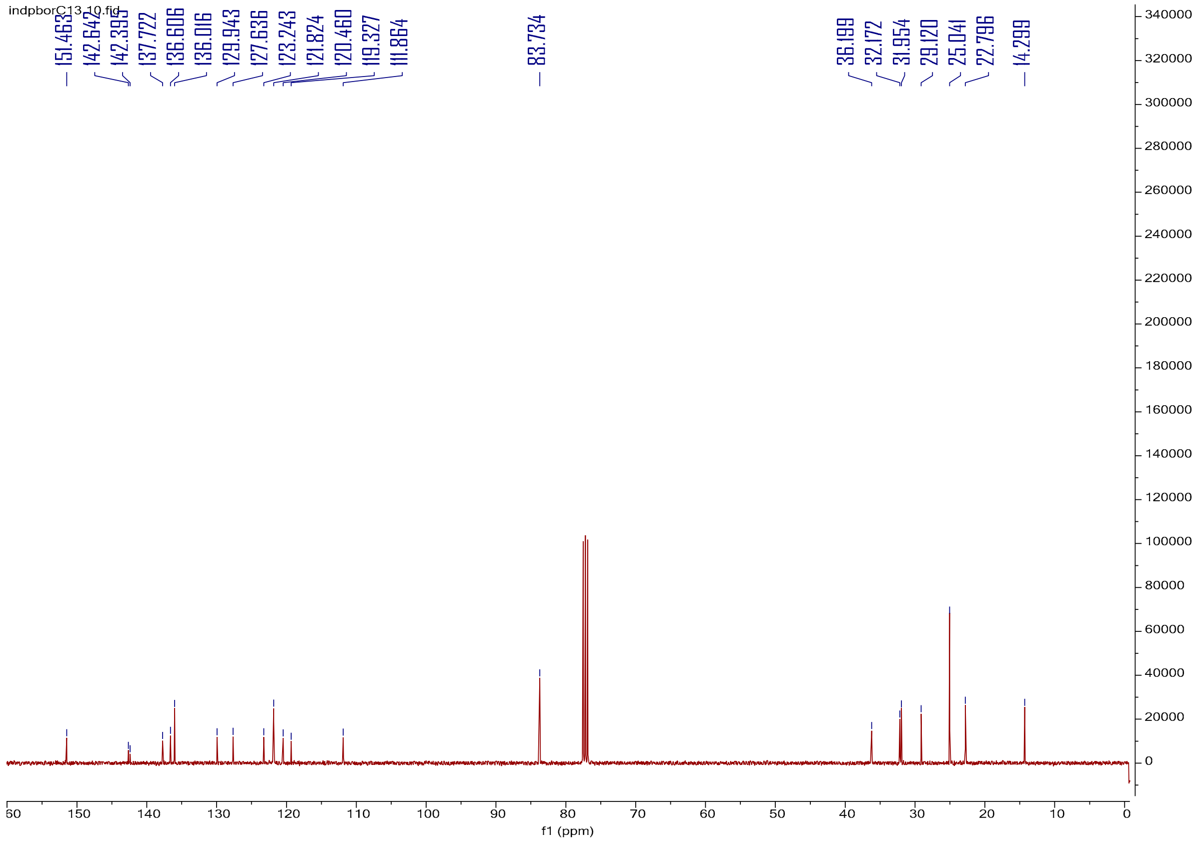

Figure S12. ^{13}C NMR spectrum of compound **9**.

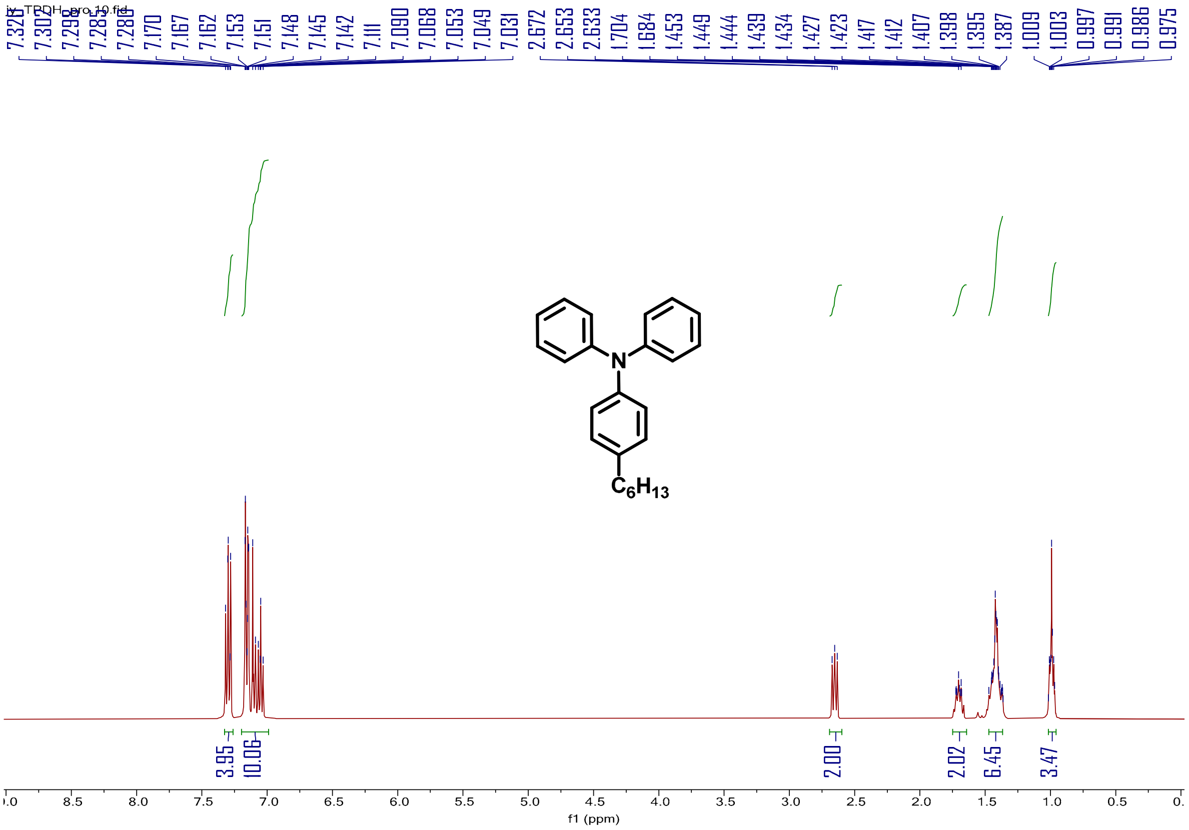

Figure S19 ^{1}H NMR spectrum of compound **10**.

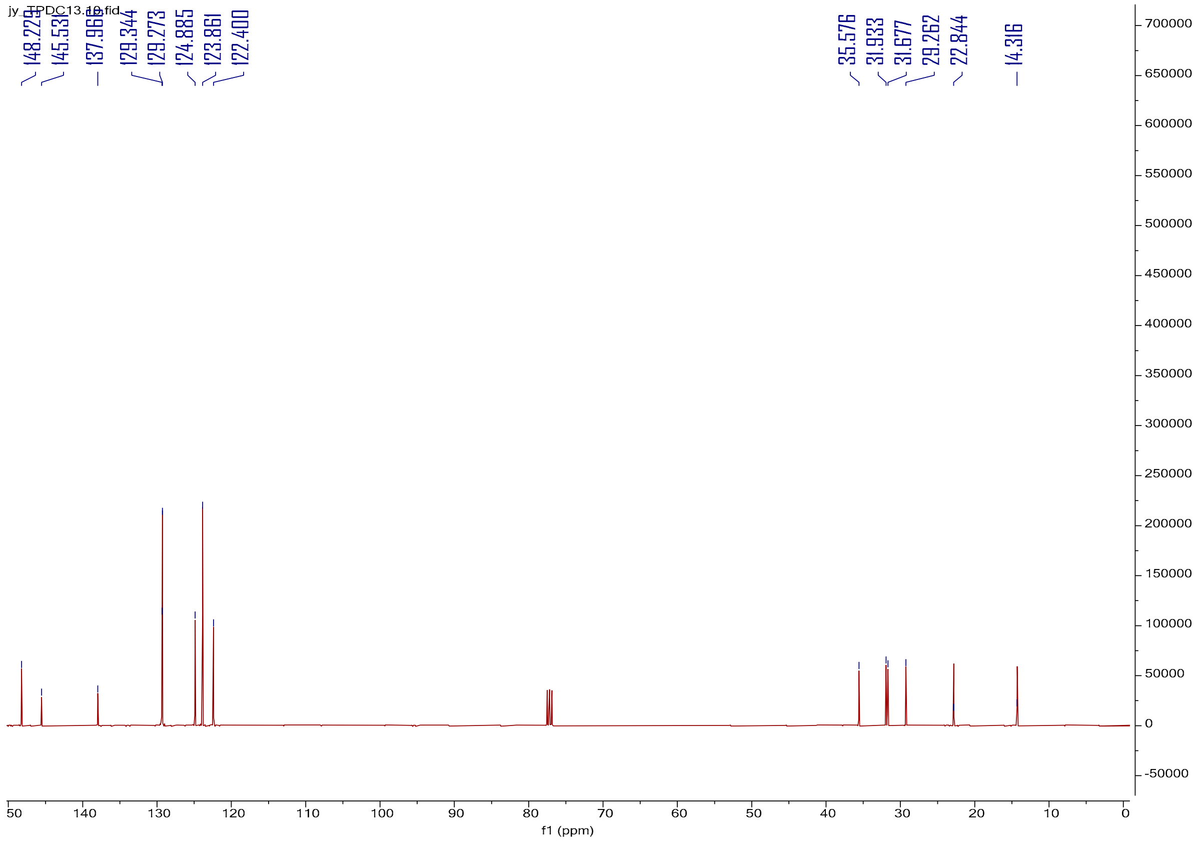

Figure S20 ^{13}C NMR spectrum of compound **10**.

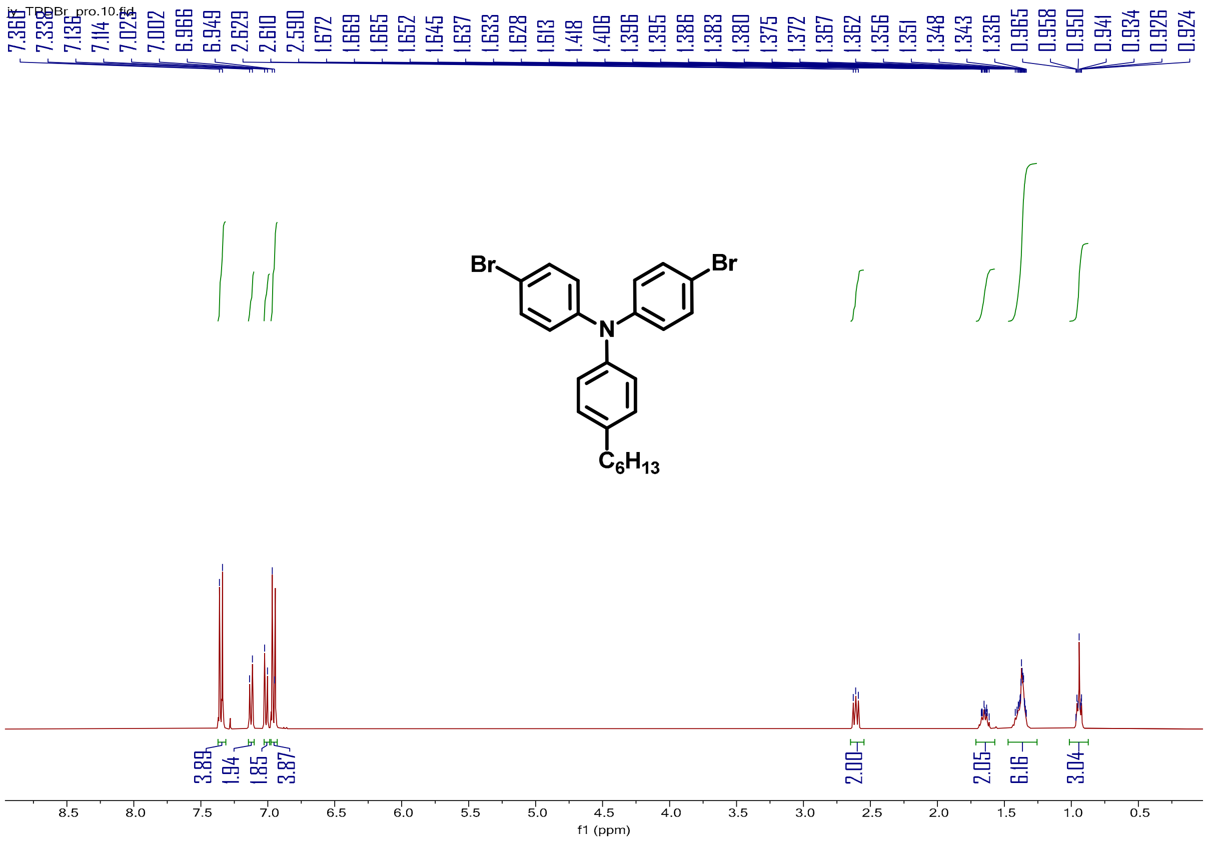

Figure 21. ^{1}H NMR spectrum of compound **11**.

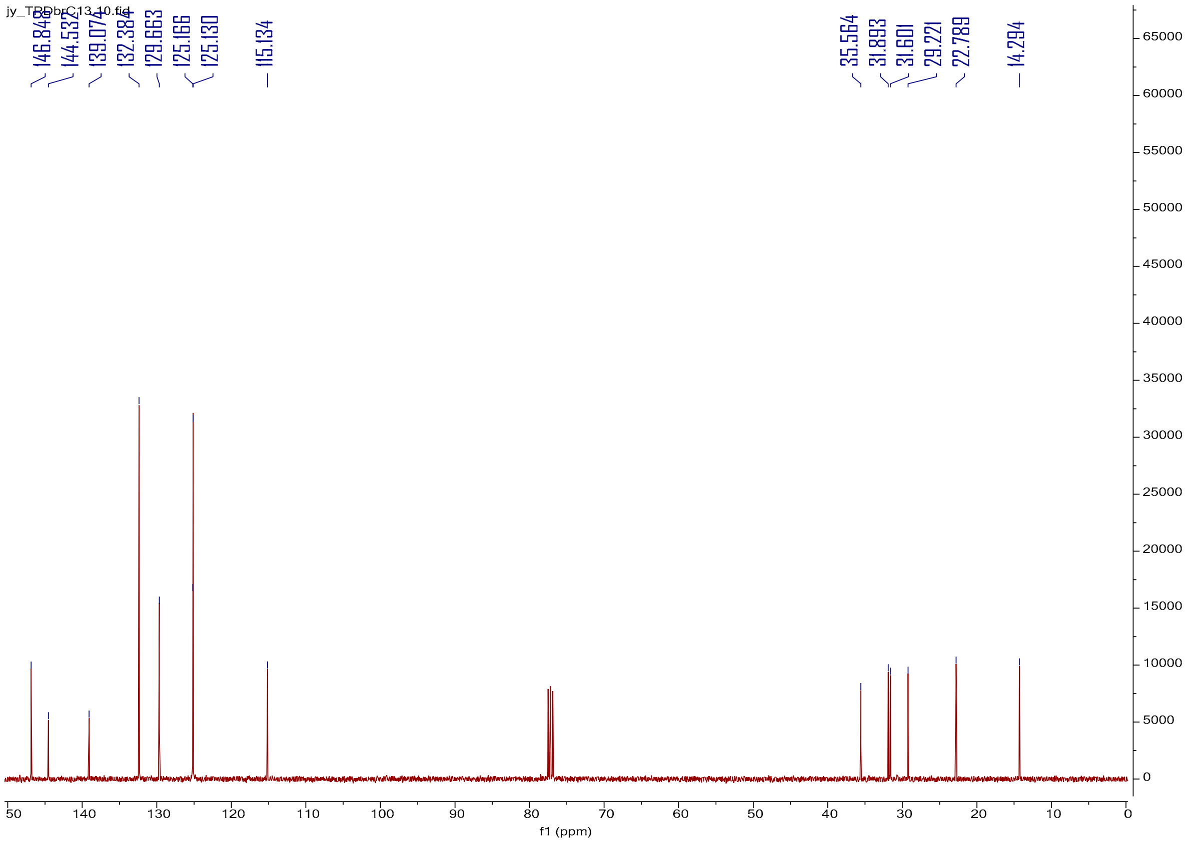

Figure S22. ^{13}C NMR spectrum of compound **11**.

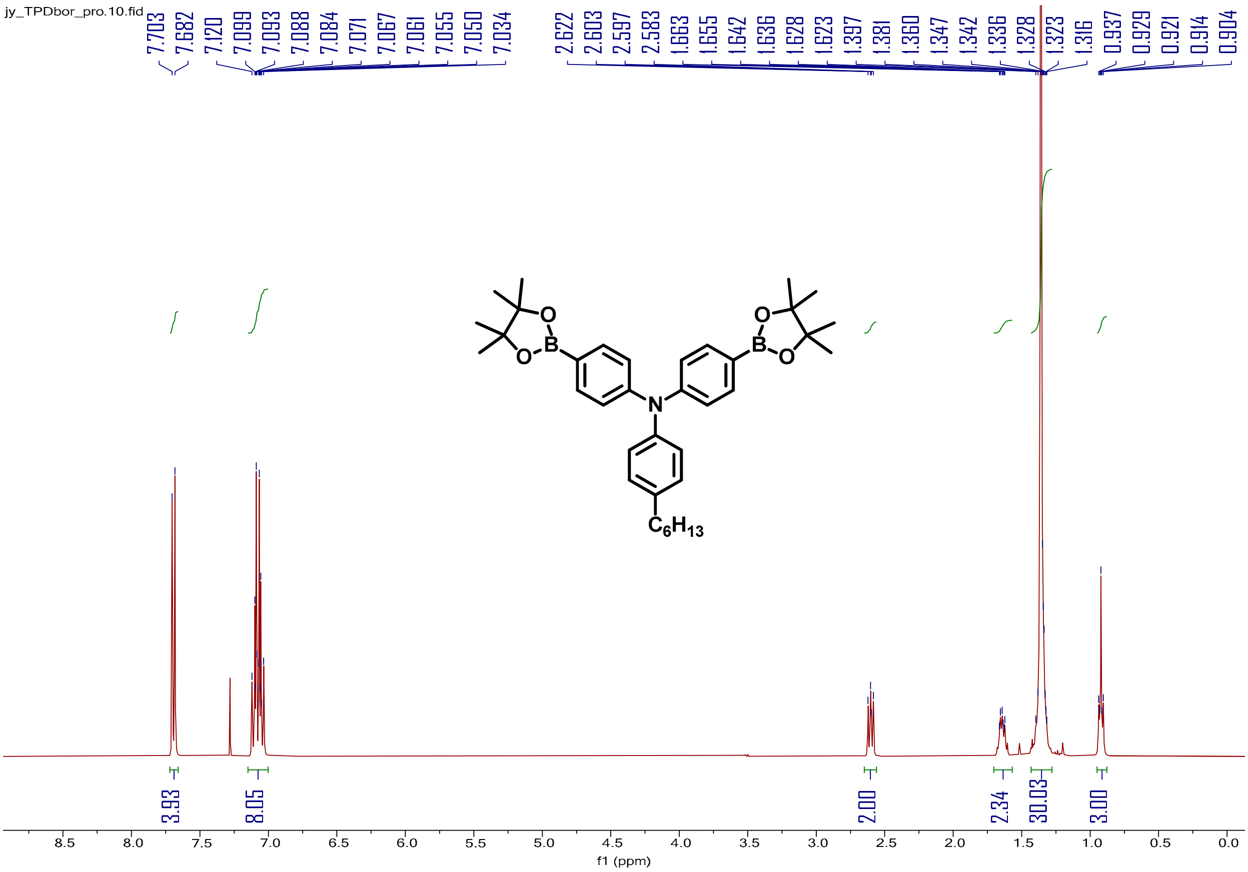

Figure S23. ^{1}H NMR spectrum of compound **12**.

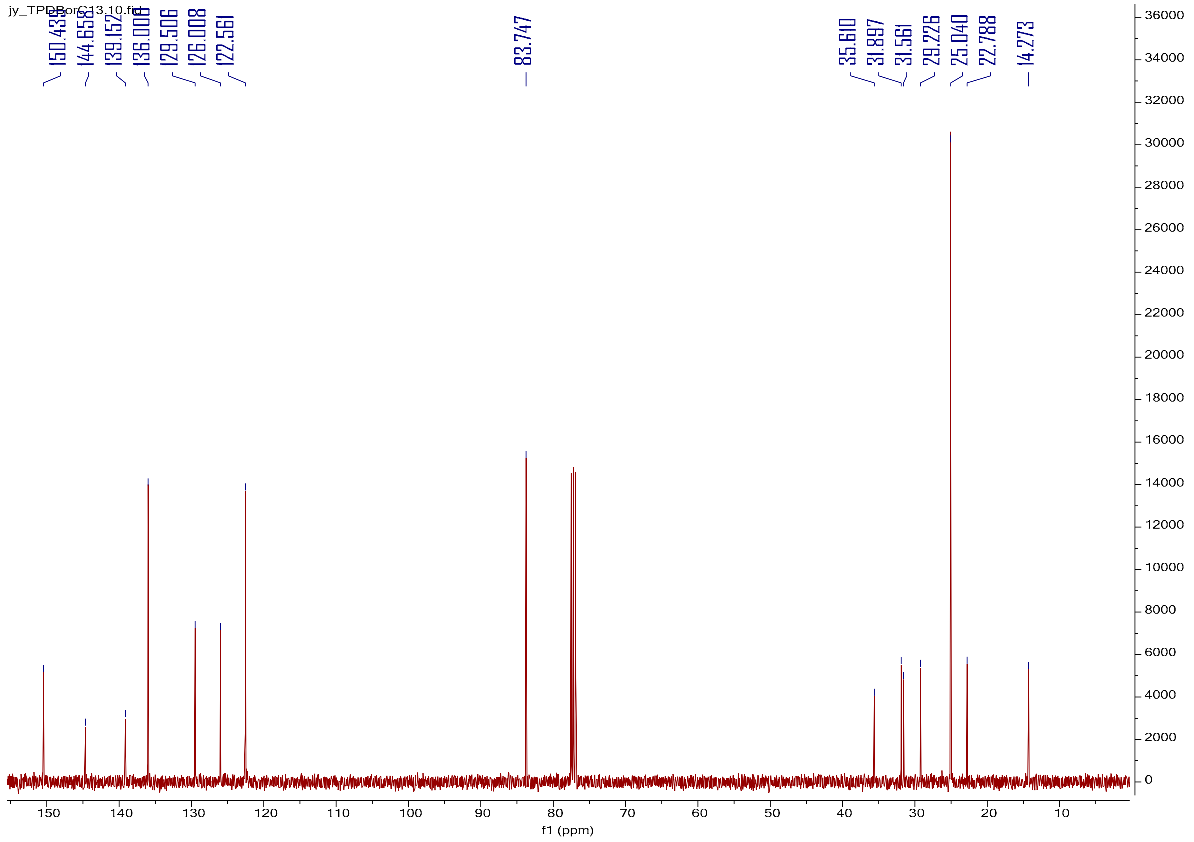

Figure S24. ^{13}C NMR spectrum of compound **12**.

Section S2. Thermal stability of PICA

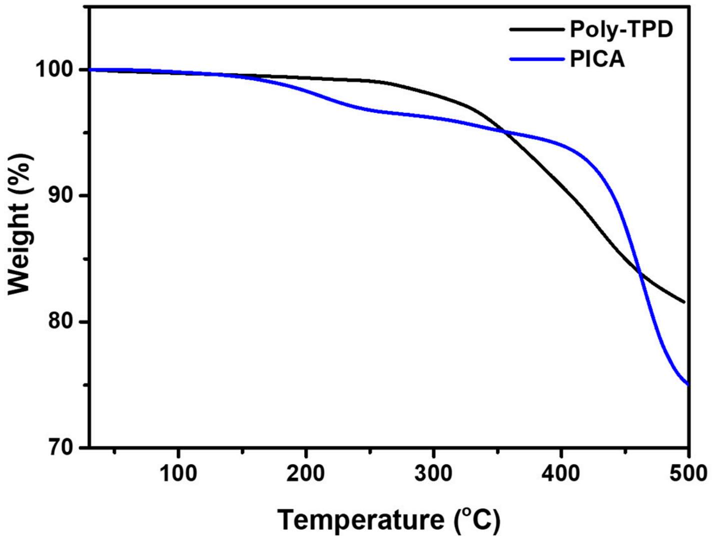

Figure S25. TGA data of PICA and Poly-TPD.

Section S3. Photoluminescence property of PICA

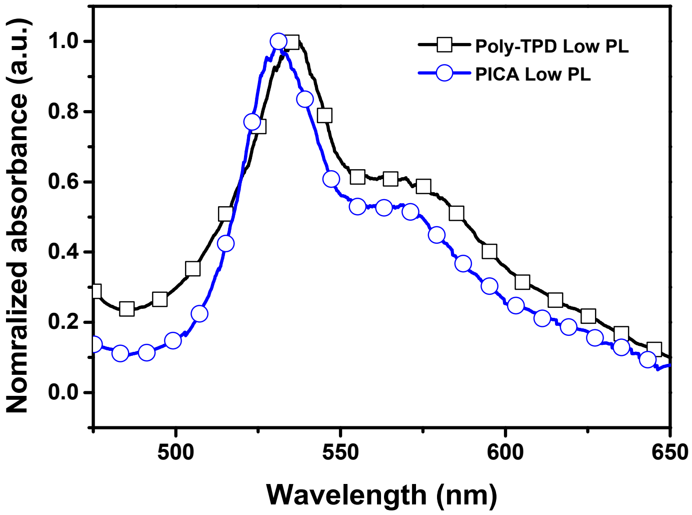

Figure S26. Photoluminescence spectra of Poly-TPD and PICA at 77K in MeTHF solutions.

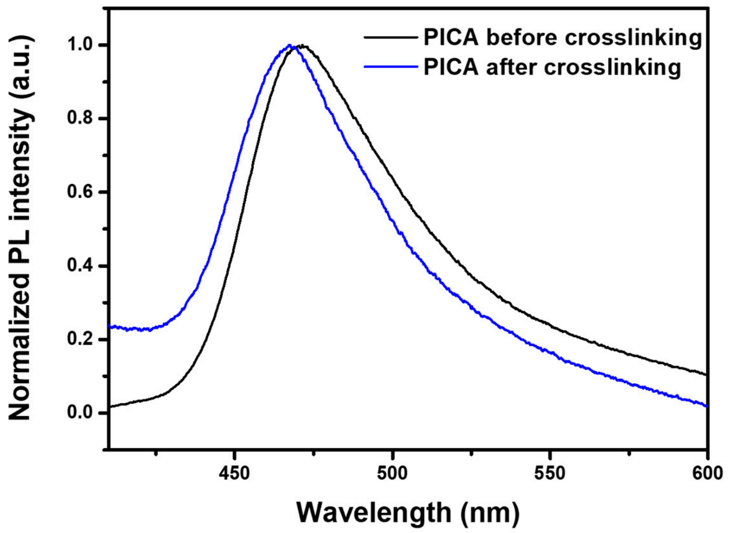

Figure S27. Photoluminescence spectra of PICA with crosslinking and non-crosslinking.

\

Section S4. Solvent resistance test of PICA with different ratios of FPA

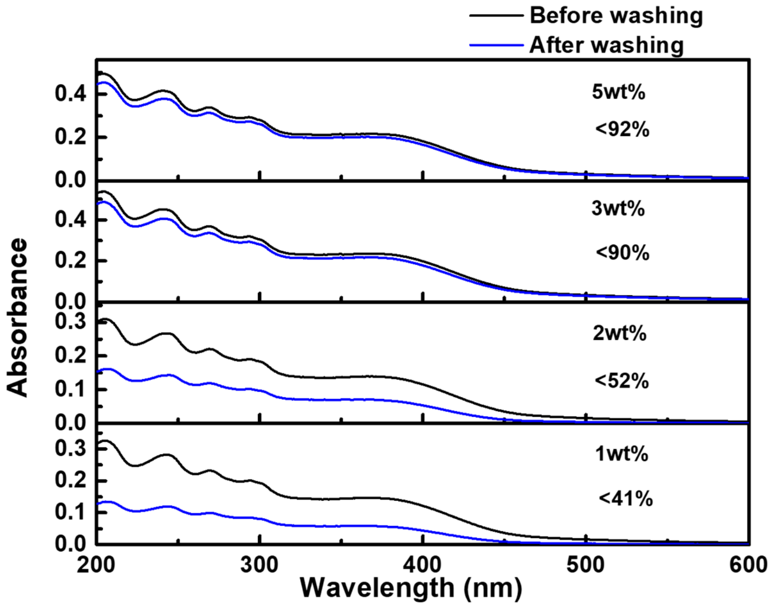

Figure S28. Solvent resistance of PICA with the different ratios of FPA.

Section S5. SCLC data of PICA and Poly-TPD

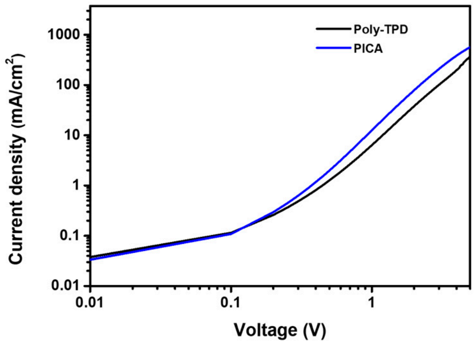

Devices	Hole mobility (cm^2 v^{-1} s^{-1})
Poly-TPD	1.3 x 10^{-5}
PICA	2.9 x 10^{-5}

Figure S29. Summary of SCLC data of hole-only devices of PICA and Poly-TPD.
